# RELA Ablation Contributes to Progression of Hepatocellular Carcinoma with TP53^R249S^ Mutation and is a Potential Therapeutic Target

**DOI:** 10.1002/advs.202500335

**Published:** 2025-09-08

**Authors:** Zhiping Wu, Zhe Wang, Diwei Zheng, Yongfang Zheng, Zhiwu Jiang, Jiang Lv, Yueqin Zhu, Heng Jia, Ziyuan Duan, Tingjie Yuan, Qiting Wu, Youguo Long, Shouheng Lin, Yao Yao, Georgia Carson, Jean P. Thiery, Kwan Man, Peng Li

**Affiliations:** ^1^ China‐New Zealand Joint Laboratory on Biomedicine and Health State Key Laboratory of Immune Response and Immunotherapy Guangdong Provincial Key Laboratory of Stem Cell and Regenerative Medicine GIBH‐HKU Guangdong‐Hong Kong Stem Cell and Regenerative Medicine Research Centre GIBH‐CUHK Joint Research Laboratory on Stem Cell and Regenerative Medicine Institute of Drug Discovery Guangzhou Institutes of Biomedicine and Health Chinese Academy of Sciences Guangzhou 510700 China; ^2^ Department of Surgery HKU‐SZH & School of Clinical Medicine Li Ka Shing Faculty of Medicine The University of Hong Kong Hong Kong SAR 999077 China; ^3^ Materials Innovation Institute for Life Sciences and Energy (MILES), HKU‐SIRI Shenzhen 518057 China; ^4^ Centre for Regenerative Medicine and Health Hong Kong Institute of Science & Innovation Chinese Academy of Sciences Hong Kong SAR China; ^5^ Houston Methodist Cancer Center Weill Cornell Medicine Houston 77030 USA; ^6^ Department of Cell Biology Yale School of Medicine New Haven 06520 USA; ^7^ Guangzhou Laboratory Guangzhou 510700 China

**Keywords:** hepatocellular carcinoma, RELA, TP53^R249S^, tumor suppressor, Wnt/β‐catenin

## Abstract

TP53 mutations are highly associated with hepatocellular carcinoma (HCC), a common and deadly cancer. However, few primary drivers in the progression of HCC with mutant TP53 have been identified. To uncover tumor suppressors in human HCC, a genome‐wide CRISPR/Cas9‐based screening of primary human hepatocytes with MYC and TP53^R249S^ overexpression (MT‐PHHs) is performed in xenografts. The screen identified *RELA* as one of the most significant genes, besides *NF2* and *CSK*, two known tumor suppressor genes (TSG) in HCC. Ablation of RELA increased the expression of genes related to cell cycling and stemness in MT‐PHHs, and induced PHHs to transform into HCC in situ in Fah‐deficient immunodeficient mice. Additionally, loss of RELA facilitated HCC metastasis via Epithelial‐Mesenchymal Transition (EMT). Clinically, low RELA expression is positively associated with poor prognosis and large tumor size in HCC patients. In terms of its underlying mechanism, reduced RELA expression promoted DVL1 expression, thereby enhancing β‐catenin nuclear translocation, and thus strengthening Wnt/β‐catenin signaling. Excitingly, betulinic acid (BetA), a RELA agonist, increased RELA activation and suppressed both growth and metastasis of hepatoma cells with TP53^R249S^ overexpression in xenografts. This study reveals RELA as a tumor suppressor in HCC with TP53^R249S^ overexpression, offering a potential therapeutic target.

## Graphical abstract

Genome‐wide CRISPR/Cas9 based screening identified RELA as a key tumor suppressor in TP53^R249S^‐mutant HCC. Its loss promotes tumorigenesis and metastasis via DVL1‐mediated Wnt/β‐catenin activation, while its agonist betulinic acid suppresses tumor progression.

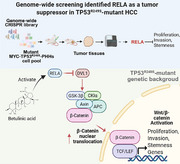

## 1 Introduction

Hepatocellular carcinoma (HCC) is the most common type of primary liver cancer (PLC) and ranks as the third leading cause of cancer‐related mortality worldwide.^[^ ^1^ ^]^ Advances in multi‐omics technologies have greatly expanded our understanding of HCC's genetic landscape, yet a breakthrough of treatment outcomes and prognosis remains elusive. With the decline of viral HCC cases due to hepatitis B vaccination and improved living conditions, an increase in HCC cases linked to alcohol abuse and non‐alcoholic steatohepatitis (NASH) is anticipated.^[^ ^2^ ^]^ Particularly in regions with high aflatoxin exposure levels such as China, where environmental and dietary factors significantly contribute to HCC risk, this necessitates a focus on identifying new risk factors and molecular mechanisms underlying these emerging HCC cases.

TP53, the most frequently defective tumor suppressor in HCC,^[^ ^3^ ^]^ plays a critical role in cell cycle arrest, apoptosis, and senescence in response to various types of stress,^[^ ^4^ ^]^ and is identified as a “trunk driver” occurring in early HCC.^[^ ^5^ ^]^ Interestingly, in more than 90% of aflatoxin‐related HCCs, the mutation pattern of TP53 is characterized as R249S.^[^ ^6^ ^]^ This predominance of R249S highlights its potential to serve as an early biomarker for hepatocarcinogenesis, underscoring the importance of studying the biological process underlying R249S somatic mutation cases in Chinese cohorts.

Previously, we generated NOD/SCID/IL2rg^−/−^/Fah^−/−^ (NSIF) mice as a potential model for recapitulating a humanized liver.^[^ ^7^ ^]^ In this model, we successfully induced a gradual transformation of primary humanized hepatocytes (PHHs) into HCC, mirroring key clinical histological characteristics. However, while the overexpression of MYC and TP53^R249S^ alone was sufficient to immortalize PHHs, it was insufficient to fully induce HCC transformation. Given that current genetic screens have yet to saturate the identification of HCC tumor suppressor genes (TSGs),^[^ ^8^ ^]^ we utilized this immortalized PHH‐derived cell line (MT‐PHHs) for a genome‐scale CRISPR screen to identify bona fide TSGs in human HCC.

RELA, also named p65, is a key transcription factor of NF‐κB signaling and is implicated in the development and progression of various cancers. For example, RELA activation has been shown to promote tumor angiogenesis and growth by increasing HIF‐1α accumulation in pancreatic cancer cells.^[^ ^9^ ^]^ Notably, NF‐κB signaling is widely reported to interact with other classical signaling pathways in the regulation of numerous biological processes,^[^ ^10^ ^]^ such as the canonical Wnt signaling pathway, which is well‐known to promote stemness, drug resistance, and metastasis in cancer cells.^[^ ^11^ ^]^ Activated NF‐κB has been found to enhance the production of Wnt ligands, β‐catenin, and β‐TrCP, contributing to cytokine storms, liver damage, and potentially fatal outcomes.^[^ ^12^ ^]^ In contrast, β‐catenin has been shown to interact with RELA, inhibiting its DNA binding activity.^[^ ^13^ ^]^ However, these effects are highly context‐dependent and require further investigation. It remains unclear whether NF‐κB/RELA and Wnt/β‐catenin interact to regulate each other in HCC development, and what specific roles these interactions may play.

Here, our screening identified RELA as a tumor suppressor in HCC, supported by clinical data and validated in both subcutaneous and long‐term orthotopic induced HCC (iHCC) models. The loss of RELA promoted Wnt/β‐catenin signaling pathway and facilitated HCC progression. Notably, we also demonstrated that betulinic acid (BetA), a RELA agonist, showed promise in inhibiting tumor growth and metastasis in hepatomas with the TP53^R249S^ mutation, potentially improving treatment outcomes and offering a novel strategic approach to liver cancer patients with TP53^R249S^ mutation.

## 2 Results

### 2.1 In Vivo Genome‐Wide CRISPR Screens Identified Potential Tumor Suppressors in Human HCC

**Figure 1:**
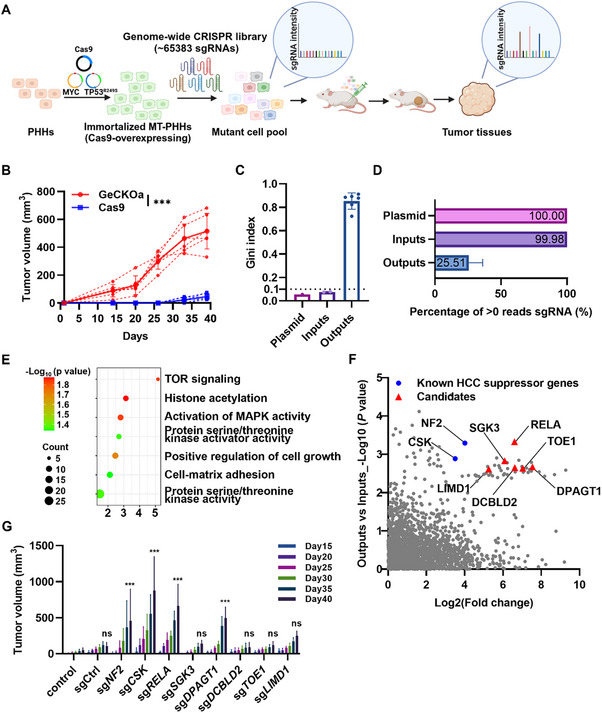
Genome‐wide CRISPR‐based screening identified candidates for HCC suppression. A) Outline of the screening strategy using human genome‐scale CRISPR/Cas9 knock‐out library (GeCKOa). B) Tumor growth curves of NSI mice transplanted with untransduced MT‐PHHs (Cas9, *n* = 6) and GeCKOa‐transduced MT‐PHHs (GeCKOa, *n* = 6). Error bars, mean ± SD. ***P ≤ 0.001, two‐way ANOVA with Tukey's multiple comparison test. Growth curves of individual tumors were also shown as dotted curves. C) Gini index representing the evenness of sgRNA read counts of Plasmid, Inputs (*n* = 2), and Outputs (*n* = 6). Error bars, mean ± SD. D) Percentage of unique sgRNAs in Plasmid, Inputs, and Outputs. Error bars for Outputs denote mean ± SD. E) Gene Ontology (GO) enrichment of the screened out sgRNA‐targeted genes. F) Identification of top candidate genes for HCC formation. Y axis represents the *P* value of 6 Output samples versus 2 Input samples calculated by MAGeCK, X axis represents the average ratios of sgRNA representations in 6 Output samples to 2 Input samples. With a Y axis cutoff of the top 50 genes and X axis cutoff greater than 10‐fold change, 8 key genes were identified, including 6 candidates (red triangles) and 2 known HCC suppressor genes (blue dots). G) Validation of the hits in F) by knocking out each target gene using its most enriched sgRNA (selected from 3 sgRNAs per genes). Average volume (*n* = 6) of tumors from Cas9‐expressed MT‐PHHs (control) and Cas9‐expressed MT‐PHHs transduced with lentivirus of a non‐targeting‐sgRNA (sgCtrl) and a subset of corresponding sgRNAs within GeCKOa library (sg*NF2*, sg*CSK*, sg*RELA*, sg*SGK3*, sg*DPAGT1*, sg*DCBLD2*, sg*TOE1*, sg*LIMD1*). Error bars, mean ± SD. ***P ≤ 0.001, two‐way ANOVA with Tukey's multiple comparison test.

To identify TSGs that inhibit tumor formation of MT‐PHHs in vivo, we transduced MT‐PHHs with a lentivirus encoding Cas9. The resulting Cas9‐expressed MT‐PHHs were then transduced with a lentiviral pool of GeCKO v2 (GeCKOa), which comprises 65383 unique single‐guide RNAs (sgRNAs) targeting 19050 human genes and 1864 miRNAs. Subsequently, 5 × 10^6^ of the mutant cells were inoculated subcutaneously into immunodeficient NOD/SCID/IL2rγ^−/−^ (NSI) mice^[^ ^14^ ^]^ (≈76 cells per sgRNA in every independent repetition) (**Figure** **1**A). Within a month, recipients of cells carrying sgRNAs targeting TSGs developed obvious subcutaneous tumors (tumor volume > 200 mm^3^) (Figure 1B). These tumors exhibited a more disorganized cellular structure with a high karyoplasmic ratio (KR) and expressed HLA and alpha‐fetoprotein (AFP), a biomarker of HCC (Figure S1A, Supporting Information), indicating they were humanized HCC. In addition, they expressed high Ki‐67 but low p21 (cyclin‐dependent kinase inhibitor) (Figure S1B–D, Supporting Information), which was consistent with their rapid expansion in vivo. Conversely, tiny lumps were observed in control group mice that received MT‐PHHs transduced with Cas9 alone (Figure 1B). They expressed AFP and Ki‐67 at low levels and highly expressed p21 (Figure S1, Supporting Information).

To identify candidate TSGs that impede tumor formation of MT‐PHHs in the genetic screening, we quantified the copy numbers of sgRNAs amplified from the extracted genomic DNA (gDNA) derived from tumors in all repetitions using high‐throughput sequencing. In parallel, we also sequenced the GeCKOa initial plasmid library (Plasmids) and the pre‐transplantation mutant MT‐PHHs (Inputs). The Gini index of tumor samples (Outputs) was significantly higher than those of Plasmid and Input samples (Figure 1C), indicating that the sgRNA distributions in Outputs was skewed, consistent with a selection process. In addition, ≈75% of sgRNAs were lost in Outputs during the screens (Figure 1D). Gene Ontology (GO) analysis uncovered that the genes targeted by sgRNAs with significantly decreased abundance in Outputs compared to Inputs were involved in the positive regulation of cell growth, histone modifications, kinase activity and intercellular interactions (Figure 1E), which contribute to malignant transformation of cells and are essential for cell survival.^[^ ^15^ ^]^ Therefore, the selected genes from the screen potentially suppress the survival advantages of MT‐PHHs.

We next compared the average ratios of sgRNAs in Outputs to that of Inputs and detected the enrichment of certain sgRNAs in Tumors. Using a cutoff of more than 10‐fold‐change in enrichment and the top 50 candidate genes identified by the MAGeCK algorithm,^[^ ^16^ ^]^ with negative controls (non‐targeting sgRNAs) included in the library design, we integrated data from 6 repetitions and identified 19 high‐hit sgRNA‐targeted genes, including the known HCC TSGs *CSK* ^[^ ^17^ ^]^ and *NF2* ^[^ ^18^ ^]^ (Figure 1F). We excluded genes that were not expressed in hepatocytes (based on NCBI‐Gene, 2024. https://www.ncbi.nlm.nih.gov/gene) and selected 8 candidate genes (*RELA*, *NF2*, *CSK*, *SGK3*, *DPAGT1*, *DCBLD2*, *TOE1* and *LIMD1*) for further functional validation in vivo (Figure 1F; Figure S2A, Supporting Information). We transplanted MT‐PHHs transduced with sgRNA targeting each candidate plus Cas9 into NSI mice and found that ablation of *NF2* or *CSK* (Figure S2B, Supporting Information) indeed accelerated HCC growth (Figure 1G), which is consistent with previous studies.^[^ ^17^, ^18^ ^]^ Of interest, MT‐PHHs transduced with sgRNA targeting RELA (sg*RELA*) and DPAGT1 (sg*DPAGT1*) also expanded more robustly than MT‐PHHs transduced with random sgRNA or Cas9 alone in vivo, suggesting that both RELA and DPAGT1 inhibited tumor growth in this model (Figure 1G). Thus, we investigated RELA and DPAGT1 as candidates for HCC suppression.

### 2.2 RELA Suppresses HCC Progression

**Figure 2:**
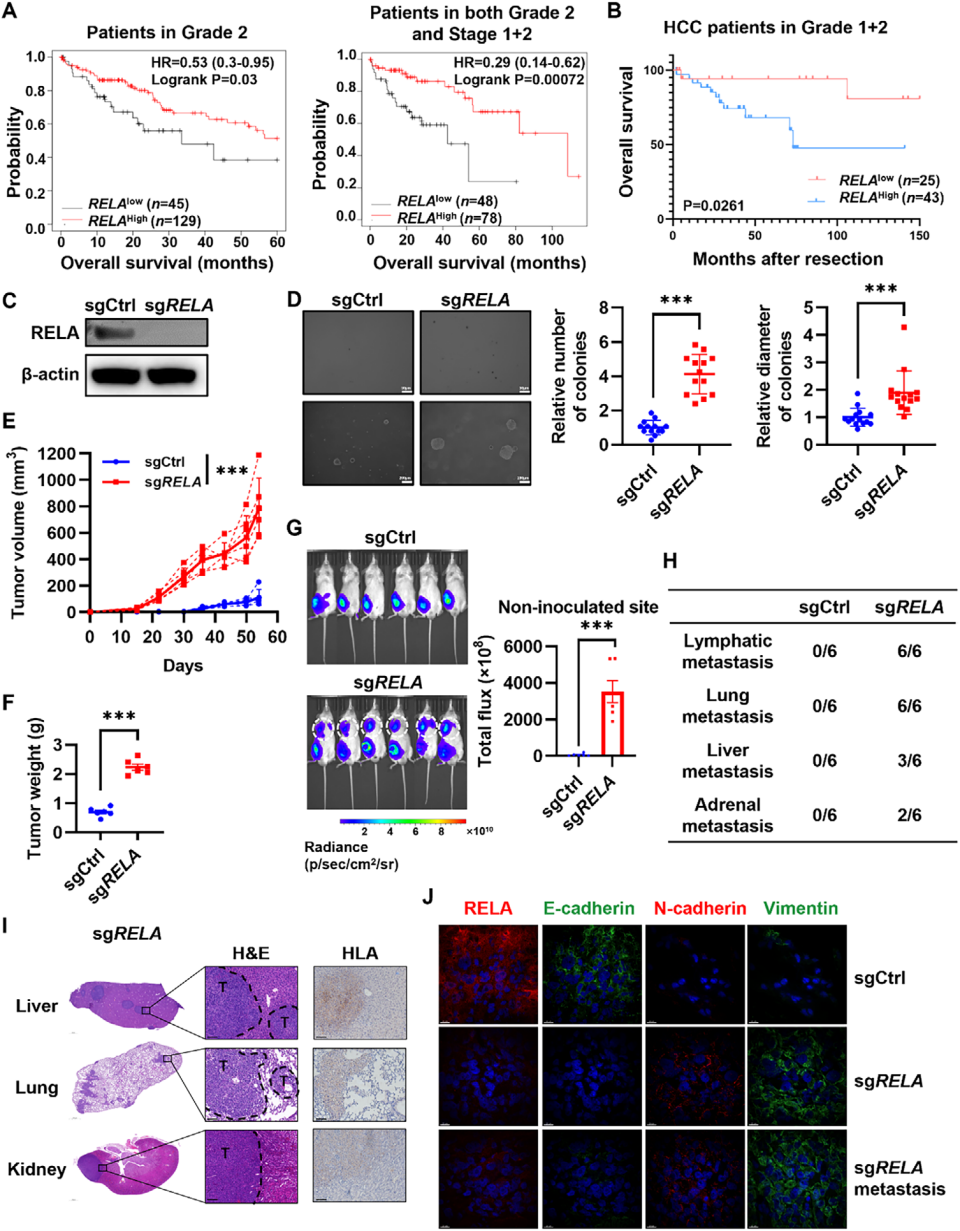
RELA deficiency promoted HCC progression. A) Kaplan–Meier analysis of the overall survival of TCGA HCC patients in Grade 2 with high (*n* = 45) or low (*n* = 129) *RELA* expression and in both Grade 2 and Stage 1+2 with high (*n* = 48) or low (*n* = 78) *RELA* expression. B) Kaplan–Meier analysis of the overall survival of clinical HCC patients in Grade 1+2 with high (*n* = 25) or low (*n* = 43) *RELA* expression. The survival curves were analyzed using the Kaplan‐Meier method, and statistical significance was determined by the log‐rank test. C) The efficacy of sgRNAs targeting *RELA* was analyzed by western blot analysis. SgCtrl, MT‐PHHs transduced with non‐targeting sgRNAs. Sg*RELA*, MT‐PHHs transduced with *RELA*‐targeting sgRNAs. D) Soft agar colony formation assay of MT‐PHHs‐sgCtrl and MT‐PHHs‐sg*RELA*. Pictures of wells are representative of three independent experiments. Pictures were obtained at 4× or 20× magnification. Scale bar, 200 or 50 µm. Relative number and relative diameter of colonies are shown. Error bars, mean ± SD. ***P ≤ 0.001, data were analyzed by two‐tailed unpaired Student's t‐test. E) The tumor growth curves of MT‐PHHs transduced with sgRELA (*n* = 6) and sgCtrl (*n* = 6). Error bars, mean ± SD. ***P ≤ 0.001, data were analyzed by two‐way ANOVA with Tukey's multiple comparison test. Growth curves of individual tumors are also shown as dotted curves. F) Tumor weight statistics are shown. Error bars, mean ± SD. ***P ≤ 0.001, data were analyzed by two‐tailed unpaired Student's t‐test. G) Bioluminescence signals were monitored in NSI mice that received MT‐PHHs‐sgCtrl or MT‐PHHs‐sg*RELA*. Cells were transduced with luciferase lentiviral expression vector pre‐transplantation. Dashed circles indicate tumor tissues grafted at non‐inoculated site. The total fluorescence intensity statistics at the non‐inoculated sites is also shown. Error bars, mean ± SD. ***P ≤ 0.001, data were analyzed by two‐tailed unpaired Student's t‐test. H) The table summarizes the occurrence of lymphatic, lung, liver and adrenal metastasis. I) Representative H&E and IHC staining images for HLA showing distinct liver, lung and kidney metastasis foci expressing HLA as shown on the right. T, Tumors. Scale bar, 1000 or 100 µm. J) Representative images of multiple immunofluorescent (IF) staining for RELA, E‐cadherin, N‐cadherin and vimentin in tumor tissue derived from MT‐PHHs‐sgCtrl and MT‐PHHs‐sg*RELA* and the resulting metastatic foci. Nuclei were counterstained with DAPI, shown in blue. Scale bars, 20 µm.

Firstly, we analyzed the relationship between *RELA* or *DPAGT1* expression and the prognosis of HCC patients using The Cancer Genome Atlas Liver Hepatocellular Carcinoma (TCGA‐LIHC) database. Interestingly, low mRNA expression of *RELA* showed a trend toward poor prognosis in all HCC patients (HR = 0.84) (Figure S3A, Supporting Information) and was significantly associated with worse outcomes in early‐stage patients or those with high differentiation (Grade 2, HR = 0.53; Grade 2 and Stage 1 + 2, HR = 0.29) (**Figure** **2**A). These findings suggest that RELA dysfunction likely represents an early event in HCC progression. In contrast, there was no evidence to demonstrate that low expression of *DPAGT1* was correlated with poor prognosis of HCC patients, even in early‐stage or well‐differentiated cases (Figure S3B,C, Supporting Information). Furthermore, to evaluate the frequency of RELA mutations in HCC patients, we analyzed the TCGA‐LIHC database on cBioPortal (http://cbioportal.org) and found that the HCC cases exhibiting deep deletion or shallow deletion of *RELA* outnumbered those with gain or amplification events (Figure S3D, Supporting Information). This observation suggests that reduced *RELA* copy number is more prevalent than increased copy number in HCC patients. Statistically, frequent genetic alterations of *RELA* accounted for 9% (31/360) of the entire HCC database (Figure S3E, Supporting Information). Among these alterations, 51.6% (16/31) of the cases were identified as missense point mutations, deep deletion or associated with low mRNA expression (Figure S3E, Supporting Information), reinforcing the potential role of *RELA* deficiency in HCC pathogenesis. We then analyzed the correlation between *RELA* expression levels and survival curves of 96 HCC patient cases from the clinical database (Table S1, Supporting Information). Consistently, low mRNA expression of *RELA* was positively associated with poor prognosis of patients with high differentiation (Grade 1 + 2, *P* = 0.0261) in that clinical cohort (Figure 2B). We next examined the correlation between *RELA* mRNA expression and clinicopathological parameters in clinical HCC patients and found that *RELA* expression was negatively associated with larger tumor size (P = 0.0177) (**Table** **1**). Taken together, these results suggested that RELA acts as a tumor suppressor in HCC progression. Therefore, we further investigated RELA's suppressive role in HCC development.

**Table 1:** Relationship between *RELA* mRNA expression and clinicopathologic characteristics in 96 HCC patients.

Feature	Number	*RELA* ^high^	*RELA* ^low^	*p value
All cases	96	47	49	
Age(years)				0.5402
≤60		21	26	
≥ 60		25	23	
Gender				0.8072
Male		36	39	
Female		11	10	
HBsAg				0.1761
Yes		37	32	
No		10	17	
Cirrhosis				0.6304
Yes		17	16	
No		30	33	
Tumor size (cm)				*0.0177
≤5		22	11	
≥ 5		25	38	
Venous infiltration				0.6810
Present		25	29	
Absent		22	20	
All cases	96	47	49	
AFP (ng/ml)				0.2543
≤400		37	33	
≥ 400		10	16	
No. of nodules (n)				0.2697
Multiple (>1)		11	17	
Single (1)		35	32	
TNM stage				0.5207
I‐II		33	31	
III‐IV		14	18	
Bil. level (µmol/L)				0.7742
Abnormal (>20)		6	8	
Normal (≤ 20)		41	41	
Extrahepatic metastasis				0.7156
Present		3	5	
Absent		43	44	
Recurrence				0.8367
Present		23	22	
Absent		23	25	

Total data from 96 HCC patients were analyzed. For the expression of *RELA* assayed by qPCR, the median expression level was used as the cutoff. Statistical analyses were carried out using the Pearson Chi‐square test. *P ≤ 0.05 was considered statistically significant.

### 2.3 RELA Deficiency Induces Tumor Growth and Metastasis of HCC

RELA regulates various detrimental effects in pancreatic cancer, such as genetic alteration, metabolic changes, acquisition of cancer stem cell properties, angiogenesis and therapy resistance.^[^ ^10^, ^19^ ^]^ To characterize the function of RELA in MT‐PHHs, we transduced a pair of sg*RELA*s into MT‐PHHs that constitutively expressed Cas9‐luciferase to ablate RELA completely (Figure 2C; Figure S4A, Supporting Information). RELA‐deficient MT‐PHHs expanded more robustly and formed more and larger colonies in soft agar culture, compared to MT‐PHHs that were transduced with non‐targeting control sgRNAs (sgCtrl) (Figure 2D; Figure S4B,C, Supporting Information). Consistently, RELA ablation upregulated the expression of genes related to the cell cycle, including *CCNB1*, *CCND1*, *CCNE1* (Figure S4D, Supporting Information),^[^ ^20^ ^]^ and genes contributing stemness like *OCT4*, *CD24*, *ANPEP*, *CD44* (Figure S4E, Supporting Information) in MT‐PHHs. We next transplanted cells subcutaneously into NSI mice and found that tumors derived from MT‐PHHs‐sg*RELA* grew faster and were larger than those in the control group (Figure 2E,F), consistent with the results in Figure 1G. Surprisingly, we also observed metastasis in MT‐PHHs‐sg*RELA*. On the final day of the observation period, luciferase fluorescent signals were detected at multiple non‐inoculated sites, including lung and upper limbs, in the MT‐PHHs‐sg*RELA* group, whereas the signals in the control group remained restricted to the original injection sites (Figure 2G). After dissection, metastatic tumors were observed in the forelimb lymph nodes and lungs of all mice in the MT‐PHHs‐sg*RELA* group, suggesting lymphatic and lung metastasis (Figure 2H). Metastases were also detected in the livers of three mice and in the adrenal glands of two mice out of six in the MT‐PHHs‐sg*RELA* group (Figure 2H). All the metastatic tumors displayed abnormal and irregular nuclear morphology compared to normal tissues and expressed human HLA (Figure 2I). Of note, primary and metastatic tumors of MT‐PHHs‐sg*RELA* upregulated N‐cadherin and vimentin expression, but downregulated E‐cadherin expression, compared to lumps of MT‐PHHs‐sgCtrl dissected from the control group (Figure 2J). In contrast with MT‐PHHs‐sg*RELA* mice, no metastatic tumors were detected in the control group (Figure 2H). Therefore, these results collectively suggest that RELA deficiency promotes the tumor growth and metastasis of MT‐PHHs in vivo.

**Figure 3:**
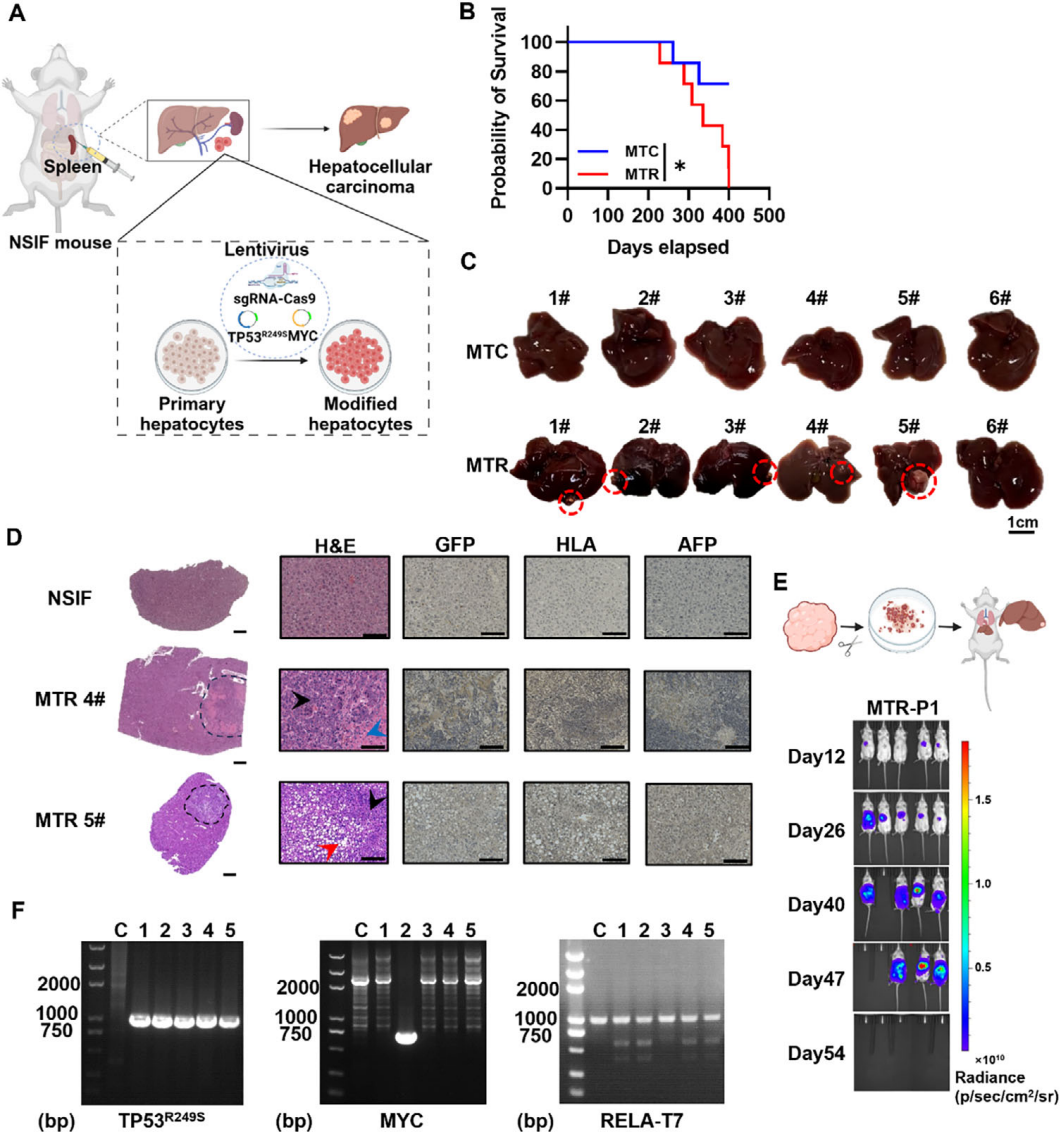
RELA deficiency promoted the transformation of PHHs into HCC with overexpression of MYC and TP53^R249S^ in situ. A) Schematic representation showing the process for induction of in situ HCC after lentiviral transduction of PHHs with TP53^R249S^ and sg*RELA*‐Cas9 overexpressing lentiviruses. B) Survival curves of mice receiving MTC (MYC, TP53^R249S^, sgCtrl‐Cas9)‐ or MTR (MYC, TP53^R249S^, sg*RELA*‐Cas9)‐transduced PHHs (*n* = 6). *P ≤ 0.05. The survival curves were analyzed using the Kaplan‐Meier method, and statistical significance was determined by the log‐rank test. C) Images of livers derived from NSIF mice receiving MTC‐ or MTR‐transduced PHHs. The *in‐situ* liver carcinomas detected in MTR group (5/6) are indicated with red dashed lines. No tumors were detected in any MTC group mouse. Scale bar, 1 cm. D) H&E staining of representative liver tissues from a control NSIF mouse and NSIF mice receiving MTR‐transduced PHHs. Scale bar, 1000 or 100 µm. Black arrows represent poorly differentiated carcinoma, red arrow represents steatohepatitic hepatocellular carcinoma, blue arrow represents necrotic tumor tissue, dashed line represents the boundary between normal and tumor tissues. IHC staining images for GFP, HLA and AFP are also shown. Scale bar, 100 µm. E) Schematic representation showing the process for secondary in situ transplantation of tumor tissues isolated from orthotopic HCC induced in NSIF mice. Bioluminescence signals were monitored periodically. The blank area represents dead mice. F) Genomic DNA (gDNA) of tumor tissues were obtained for PCR amplification using forward primers that recognize TP53^R249S^, MYC, or RELA‐T7 and reverse primers specific for eGFP. Representative agarose gel electrophoresis image showing the digestion of these PCR products by TP53^R249S^ or MYC and reverse primers specific for eGFP, and T7 Endonuclease I (a mismatch‐specific nuclease that cleaves heteroduplex DNA at mutation sites). Lane 1: DNA ladder (marker). Lane 2: negative control. Lanes 3–7: Genomic DNA (gDNA) of tumor tissues. DNA sequencing confirmed the translation of exogenous MYC, TP53^R249S^ or sg*RELA*.

To validate whether RELA deficiency facilitates the transformation of primary humanized hepatocytes (PHHs) into HCC with MYC and TP53^R249S^ overexpression in situ, we transduced PHHs with a mixture of lentivirus overexpressing MYC and TP53^R249S^, along with either a pair of non‐targeting‐sgRNAs (MTC) or *RELA*‐sgRNAs coupled with Cas9 (MTR). These modified cells were then intrasplenically transplanted into NSIF mice to establish a humanized, long‐term orthotopic spontaneous mouse model (**Figure** **3**A). A gradual health decline, culminating in mortality within 400 days, was observed in NSIF mice receiving MTR‐PHHs transplantation, whereas MTC‐PHHs‐transplanted mice exhibited significantly prolonged survival (Figure 3B). Following dissection, white nodules were found in the liver of 5 recipients of MTR‐PHHs, but none were observed in the control group (Figure 3C). Compared to NSIF mice with Fah knockout‐induced liver injury,^[^ ^7^ ^]^ the histological morphology of the humanized livers from surviving recipients of MTR‐PHHs was distinct (Figure 3D). Specifically, H&E analysis confirmed that the white nodules represented tumor tissues, demonstrating features of steatohepatitis combined with poorly differentiated carcinoma and necrotic tumor tissue (Figure 3D; Figure S5A, Supporting Information). IHC images for AFP, HLA and GFP also indicated the histological characteristics of orthotopic HCC tumors (Figure 3D; Figure S5A, Supporting Information). Moreover, upon secondary orthotopic transplantation of primary tumor chunks, the tumor tissues rapidly engrafted onto the livers of NSI mice and grew robustly, ultimately resulting in the mortality of the mice (Figure 3E). Collectively, this evidence demonstrates that the loss of RELA results in HCC progression.

### 2.4 RELA Acts as a Tumor Suppressor in a TP53^R249S^ Mutation‐Dependent Manner

DNA sequencing results confirmed the transduction of TP53^R249S^ (Figure 3F; Figure S5B, Supporting Information) as well as the Cas9‐resulted cleavage of RELA (Figure 3F) in all the transformed HCC tumors, while, interestingly, the exogenous MYC overexpression was only observed in one tumor (1/5) (Figure 3F). These results suggest that only TP53^R249S^ overexpression and RELA deletion are essential for transforming PHHs into HCC in situ, whereas MYC appears to be dispensable in this process.

To confirm this hypothesis, we transduced TP53^R249S^ into the HepG2 cell line, a hepatoma cell line with an intact TP53,^[^ ^21^ ^]^ and then ablated RELA in both HepG2‐WT and HepG2‐TP53^R249S^. As expected, we found that RELA deletion significantly promoted cell proliferation in HepG2‐TP53^R249S^ cells, but not in HepG2‐WT cells (Figure S6A, Supporting Information). In addition, RELA ablation did not alter the colony formation capacity of HepG2‐WT cells (Figure S6B, Supporting Information), while its deletion did significantly increase both the number and size of colonies in HepG2‐TP53^R249S^ cells (Figure S6C, Supporting Information). In line with the in vitro data, RELA‐deficient HepG2‐WT cells did not exhibit an expansion advantage over control cells in xenografts (Figure S6D,E, Supporting Information), whereas RELA ablation enhanced tumor growth of HepG2‐TP53^R249S^ in vivo (Figure S6D,E, Supporting Information). To exclude the possibility of MYC dependence, we also analyzed RELA's role in HepG2‐WT and HepG2‐MYC cells. In contrast with the TP53^R249S^ results, RELA deletion did not enhance cell proliferation or colony formation capacity in either HepG2‐WT or HepG2‐MYC cells (Figure S6F,G, Supporting Information). These results demonstrate that RELA plays a tumor‐suppressive role in a TP53^R249S^ mutation‐dependent manner.

### 2.5 RELA Deficiency Promotes the Metastasis of HCC via EMT Program

**Figure 4:**
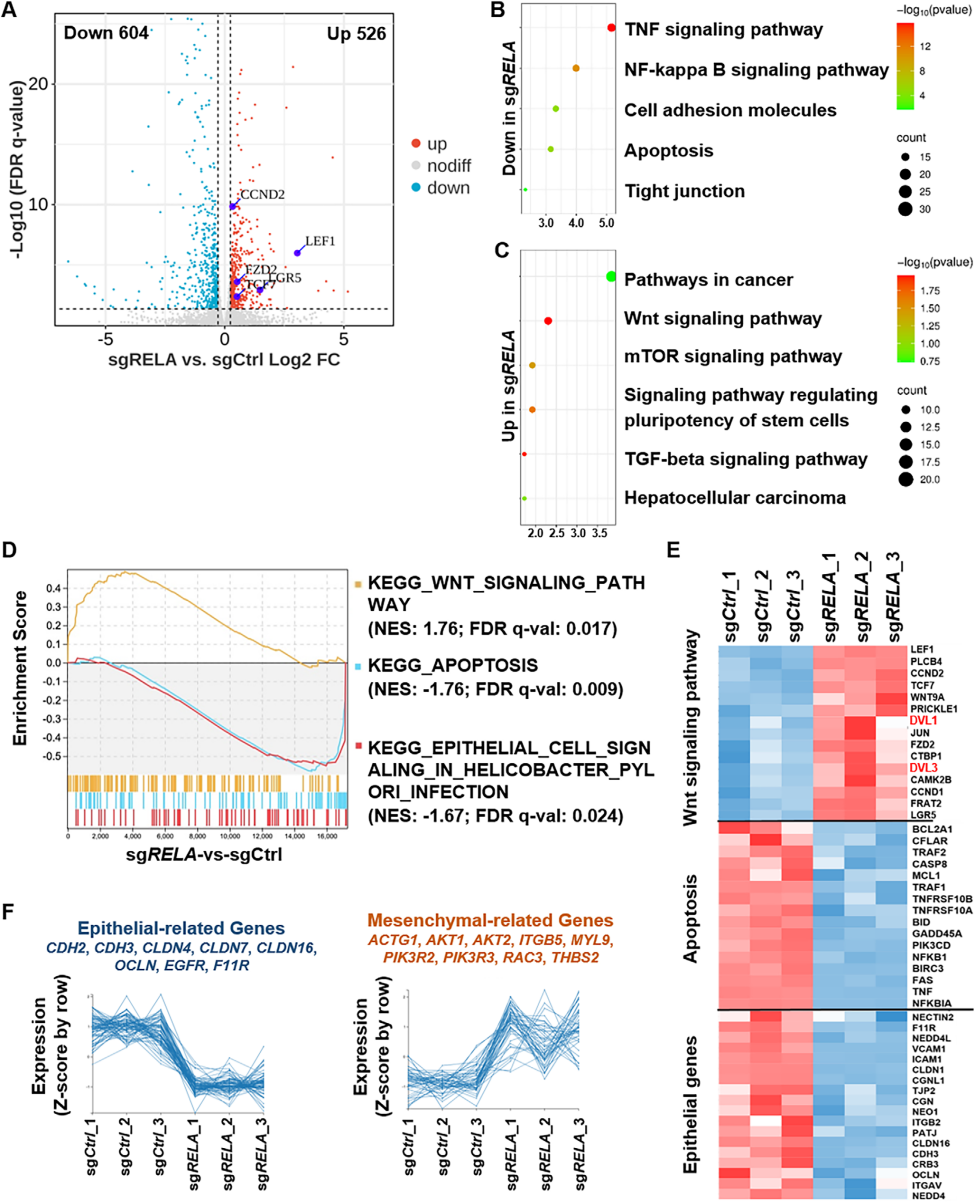
Comprehensive RNA‐seq analysis revealed the global transcriptome in MT‐PHHs upon RELA deletion. A) Volcano plots depicting differentially expressed genes (DEGs) in MT‐PHHs‐sg*RELA* compared to MT‐PHHs‐sgCtrl, with upregulated genes (Log2FC ≥ 0.26 and FDR q‐value ≤ 0.05) marked in red, downregulated genes (Log2FC ≤ ‐0.26 and FDR q‐value ≤ 0.05) in blue, and non‐differentially expressed genes in gray. Wnt signaling pathway genes (*LEF1*, *LGR5*, *TCF7*, *FZD2*, *CCND2*) are highlighted in purple. B) Kyoto Encyclopedia of Genes and Genomes (KEGG) analysis of the pathways enriched with downregulated genes in MT‐PHHs‐sg*RELA*. C) KEGG analysis of the pathways enriched with upregulated genes in MT‐PHHs‐sg*RELA*. D) Gene set enrichment analysis (GSEA) of KEGG Wnt signaling pathway, KEGG apoptosis, and KEGG epithelial cell signaling in helicobacter pylori infection in MT‐PHHs‐sgCtrl and MT‐PHHs‐sg*RELA*. NES, normalized enrichment score. E) Heat map showing the values of selected genes that had differential expression in MT‐PHHs‐sgCtrl and MT‐PHHs‐sg*RELA* (*n* = 3). Genes were classified into Wnt signaling pathway, cell apoptosis or epithelial characteristics. F) mRNA expression levels of epithelial‐related and mesenchymal‐related genes in 3 independent samples of MT‐PHHs‐sgCtrl and MT‐PHHs‐sg*RELA* are shown.

To dissect the effects of RELA deletion on the global transcriptome, we conducted RNA‐sequencing (RNA‐seq) analysis and compared the transcriptional profiles between MT‐PHHs‐sgCtrl and MT‐PHHs‐sg*RELA* cultured in vitro. RNA‐seq analysis identified 604 downregulated and 526 upregulated differentially expressed genes (DEGs) in MT‐PHHs‐sg*RELA* compared to MT‐PHHs‐sgCtrl (**Figure** **4**A). Kyoto Encyclopedia of Genes and Genomes (KEGG) analysis showed that downregulated DEGs in MT‐PHHs‐sg*RELA* were mainly associated with the pathways related to TNF signaling, NF‐κB signaling, apoptosis and epithelial formation, while upregulated DEGs were highly enriched in the pathways related to hepatocellular carcinoma, Wnt signaling and TGF‐β signaling (Figure 4B,C). Gene set enrichment analysis (GSEA) and heatmap analysis also revealed that Wnt‐related genes were positively correlated with RELA knockout, whereas apoptosis and epithelial cell‐related genes exhibited a negative correlation (Figure 4D,E).

Of note, the downregulation of the cell adhesion molecules (CAMs) and the upregulation of the Wnt and TGF‐β signaling pathways in MT‐PHHs‐sg*RELA* suggested a promoted Epithelial‐Mesenchymal Transition (EMT) program (Figure 4B–E).^[^ ^22^ ^]^ Transcriptionally, the MT‐PHHs‐sg*RELA* exhibited a significant EMT profile (Figure 4F). As EMT is required for metastasis,^[^ ^23^ ^]^ these results were in line with the findings that RELA ablation promoted metastasis of MT‐PHHs in xenografts (Figure 2G–J).

EMT enables stationary carcinoma cells to acquire the capacity for invasion and dissemination during metastasis,^[^ ^24^ ^]^ prompting further investigation of EMT signatures in MT‐PHHs‐sg*RELA*. First, we compared cellular morphology between MT‐PHHs‐sgCtrl and MT‐PHHs‐sg*RELA*. As expected, MT‐PHHs lost polarity and exhibited “Indian file” morphology upon RELA ablation (Figure S7A, Supporting Information). Furthermore, MT‐PHHs‐sg*RELA* exhibited significantly enhanced migration and invasion capacities compared to controls, as evidenced by Transwell assays (Figure S7B, Supporting Information). We also measured the markers during EMT and found that mesenchymal‐related markers, including Zeb1, vimentin, and Snail1, were increased, while E‐cadherin, an epithelial marker, was decreased in MT‐PHHs‐sg*RELA*, compared to controls (Figure S7C, Supporting Information). Indeed, EMT has been demonstrated to disrupt and reverse the apical‐basal polarity axis.^[^ ^25^ ^]^ When cultured in 3D spheroids, both MT‐PHHs‐sgCtrl and MT‐PHHs‐sg*RELA* formed actin‐enriched tubules (Figure S7D, Supporting Information). However, an apical‐basal polarity was maintained in the 3D spheroids formed by MT‐PHHs‐sgCtrl, characterized by the localization of the lateroapical protein ZO‐1 on the apical membrane (Figure S7D, Supporting Information). In contrast, MT‐PHHs‐ sg*RELA* lost this apical‐basal polarity (Figure S7D, Supporting Information). Taken together, these results indicate that EMT program is initiated in MT‐PHHs upon RELA loss, thereby inducing tumor metastasis.

### 2.6 RELA Negatively Regulates Activation of the Wnt/β‐Catenin Signaling Pathway

**Figure 5:**
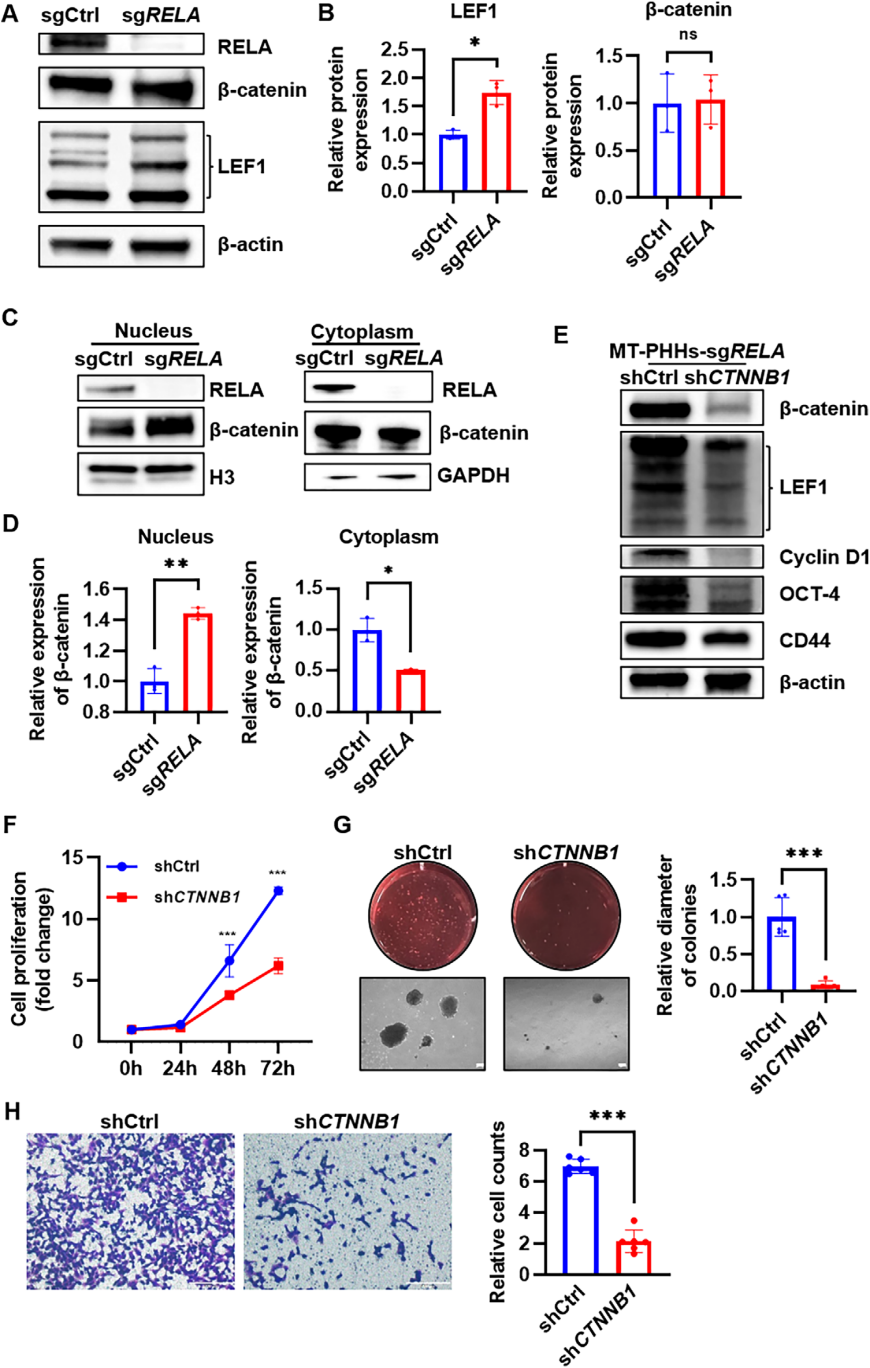
RELA ablation maintained HCC stemness through Wnt/β‐catenin signaling. A) The total protein expression of β‐catenin and its downstream LEF1 were measured by western blot. B) Relative protein expressions are shown. Error bars, mean ± SD. *P ≤ 0.05, ns > 0.05, data were analyzed by two‐tailed paired Student's t‐test. C) The nuclear and cytoplasmic expression of β‐catenin and RELA in MT‐PHHs‐sgCtrl and MT‐PHHs‐sg*RELA* were examined by western blot analysis. H3 and GAPDH were used as the nuclear and cytoplasmic reference, respectively. D) Relative protein expressions are shown. Error bars, mean ± SD. *P ≤ 0.05, **P ≤ 0.01, data were analyzed by two‐tailed paired Student's t‐test. E) Western blot analysis of protein levels of β‐catenin, LEF1, cyclin D1, OCT‐4 and CD44 in shCtrl‐ and sh*CTNNB1*‐transduced MT‐PHHs‐sg*RELA*. F) Representative proliferation curves for shCtrl‐ or sh*CTNNB1*‐transduced MT‐PHHs‐sg*RELA*. Error bars, mean ± SD. ***P ≤ 0.001, data were analyzed by two‐way ANOVA with Tukey's multiple comparison test. G) Soft agar colony formation assay of shCtrl‐ or sh*CTNNB1*‐transduced MT‐PHHs‐sg*RELA*. Images are representative of three independent experiments. Full‐view images show the number of colonies, and the 4× magnification images indicate the size of the colonies. Scale bar, 200 µm. Relative diameter of colonies is shown. Error bars, mean ± SD. ***P ≤ 0.001, data were analyzed by two‐tailed paired Student's t‐test. H) Cell invasion of shCtrl‐ or sh*CTNNB1*‐transduced MT‐PHHs‐sg*RELA* were estimated using Transwell assay and quantified by relative counts of crystalline violet‐stained cells. Scale bar, 200 µm. Error bars, mean ± SD. ***P ≤ 0.001, data were analyzed by two‐tailed paired Student's t‐test. Pictures of wells are representative of three independent experiments.

Since the Wnt/β‐catenin signaling pathway is one of the intrinsic regulators of proliferation and stemness in HCC, contributing to HCC progression and metastasis,^[^ ^26^ ^]^ we focused on the transcriptionally upregulated Wnt signaling pathway in MT‐PHHs‐sg*RELA*. Notably, MT‐PHHs‐sg*RELA* exhibited higher expression of *LEF1*, *TCF7*, *WNT9A*, *DVL1*, *DVL3*, *CCND1* and *LGR5*, compared to control cells (Figure 4A,E), suggesting that the canonical Wnt/β‐catenin signaling pathway was activated^[^ ^26a^ ^]^ and may drive HCC progression upon RELA ablation. Indeed, the downstream targets of Wnt/β‐catenin, including *LEF1*, *TCF7*, *JUN*, *APC*, *CCND1*, *CD44* and *MMP7*, were all elevated in MT‐PHHs upon RELA ablation (**Figure** **5**A,B; Figures S4D,E and Figure S8A,B, Supporting Information). Though neither the overall mRNA or protein levels of β‐catenin changed (Figure 5A,B; Figure S8C, Supporting Information), interestingly, the accumulation of β‐catenin in the nucleus rather than cytoplasm was increased in MT‐PHHs‐sg*RELA* (Figure 5C,D), thereby enhancing the activation of Wnt/β‐catenin signaling. To examine whether RELA deletion promoted the expansion and stemness of MT‐PHHs through activating Wnt/β‐catenin signaling, we transduced shRNAs targeting *CTNNB1* (sh*CTNNB1*) into MT‐PHHs‐sg*RELA* to knock down β‐catenin (Figure 5E). Indeed, MT‐PHHs‐sg*RELA*‐sh*CTNNB1* (MTR‐PHHs‐sh*CTNNB1*) expanded significantly slower than MT‐PHHs‐sg*RELA*‐shCtrl (MTR‐PHHs‐shCtrl) (Figure 5F), and the colony formation capacity was also attenuated (Figure 5G). Moreover, the mRNA levels of stemness markers (*OCT4*, *ANPEP*, *CD24*, *CD44* and *CD90*) were reduced in MTR‐PHHs upon the knockdown of *CTNNB1* (Figure S8D, Supporting Information). Consistently, the protein levels of OCT‐4, Cyclin D1 and CD44 were also downregulated in MTR‐PHHs‐sh*CTNNB1* (Figure 5E). Furthermore, β‐catenin inhibition also diminished the invasion ability of MT‐PHHs‐sg*RELA* (Figure 5H). Altogether, these results demonstrate that RELA deficiency promotes the expansion capacity and cell stemness of MT‐PHHs through activation of the Wnt/β‐catenin signaling pathway.

### 2.7 RELA Restrains the Nuclear Translocation of β‐Catenin by Repressing DVL1 Expression

**Figure 6:**
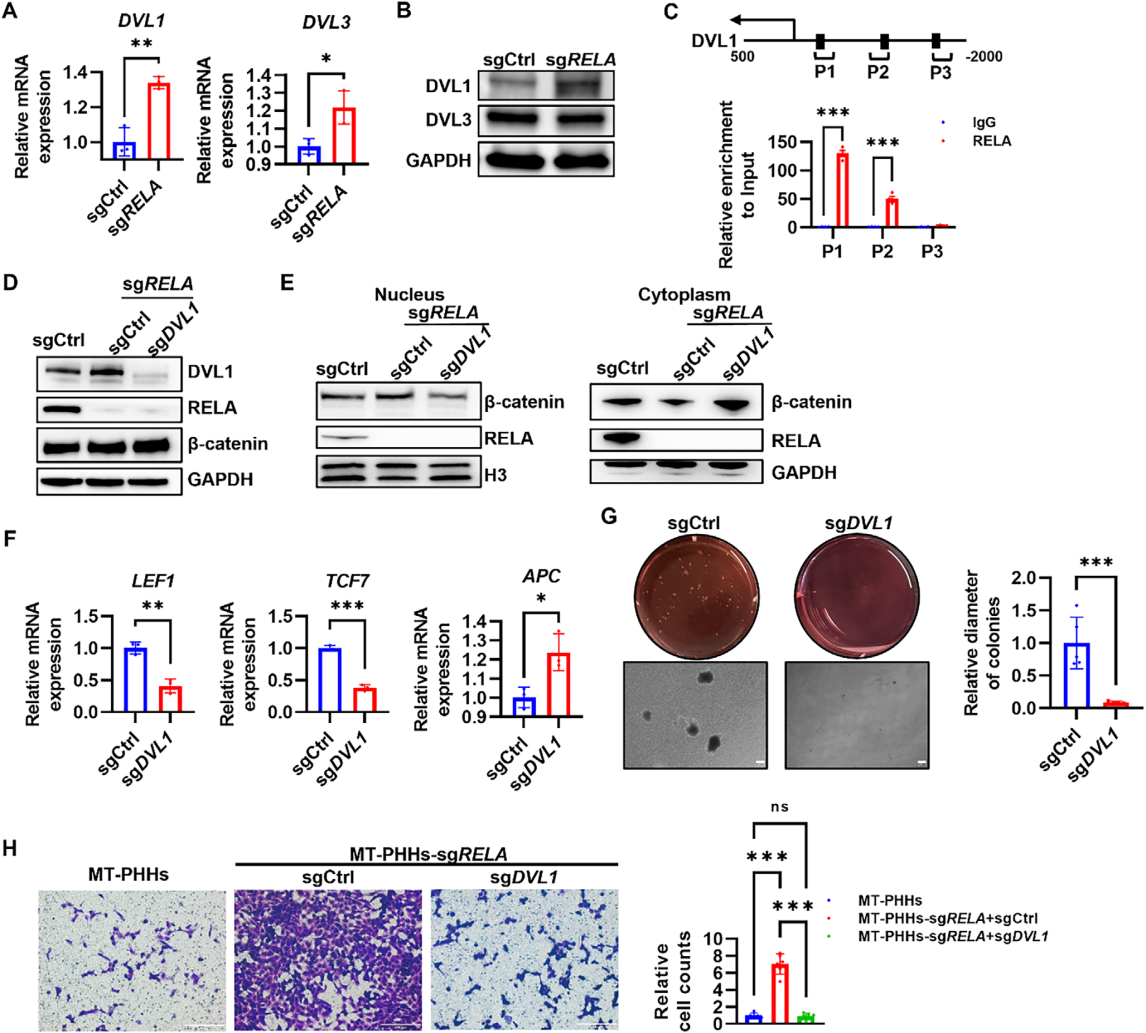
RELA downregulated DVL1 expression, reducing the nuclear translocation of β‐catenin. A) Relative mRNA levels of *DVL1* and *DVL3* in MT‐PHHs‐sgCtrl and MT‐PHHs‐sg*RELA*, based on quantitative RT‐PCR. The results represent mean ± SD. *P ≤ 0.05; **P ≤ 0.01, data were analyzed by two‐tailed paired Student's t‐test. B) Western blot analysis of DVL1 and DVL3 protein levels in MT‐PHHs‐sgCtrl and MT‐PHHs‐sg*RELA*. C) Quantitative validation by CUT&Tag‐qPCR demonstrated *RELA* enrichment at the *DVL1* promoter compared to Input control (IgG). Error bars, mean ± SD. ***P ≤ 0.001, data were analyzed by two‐tailed unpaired Student's t‐test. D) Western blot analysis of DVL1, RELA and β‐catenin protein levels in MT‐PHHs‐sgCtrl and MT‐PHHs‐sg*RELA*. E) The nuclear and cytoplasmic expression of β‐catenin and RELA in MT‐PHHs and sgCtrl‐ or sg*DVL1*‐transduced MT‐PHHs‐sg*RELA* were examined by western blot analysis. H3 or GAPDH was used as the nuclear and cytoplasmic reference, respectively. The data are presented as the mean ± SD. F) Relative mRNA levels of *LEF1*, *TCF7* and *APC* in sgCtrl‐ or sg*DVL1*‐transduced MT‐PHHs‐sg*RELA*, based on quantitative RT‐PCR. The results represent mean ± SD. *P ≤ 0.05; **P ≤ 0.01; ***P ≤ 0.001, data were analyzed by two‐tailed paired Student's t‐test. G) Soft agar colony formation assay of sgCtrl‐ or sg*DVL1*‐transduced MT‐PHHs‐sg*RELA*. The images are representative of three independent experiments. Full‐view images show the number of colonies, and the 4× magnification images indicate the size of the colonies. Scale bar, 200 µm. Relative diameter of colonies is shown. The results represent mean ± SD. ***P ≤ 0.001, data were analyzed by two‐tailed paired Student's t‐test. H) Cell invasion of MT‐PHHs and sgCtrl‐ or sg*DVL1*‐transduced MT‐PHHs‐sg*RELA* were estimated using Transwell assay and quantified by relative counts of crystalline violet‐stained cells. Scale bar, 200 µm. The results represent mean ± SD. ***P ≤ 0.001, data were analyzed by two‐tailed paired Student's t‐test. Pictures of wells are representative of three independent experiments.

To explore how RELA inhibits the Wnt/β‐catenin signaling pathway, we analyzed the RNA‐seq data and found that *DVL1* and *DVL3*, members of the Disheveled (DVL) family (that promote the disassembly of the β‐catenin destruction complex through the subsequent recruitment of Axin^[^ ^26a^ ^]^), were upregulated in MT‐PHHs‐sg*RELA*, compared to control cells (Figure 4E). Consistently, qRT‐PCR also confirmed their increased mRNA levels (**Figure** **6**A). However, the protein levels of DVL1 were significantly increased, whereas DVL3 remained unchanged in MT‐PHHs upon RELA ablation (Figure 6B). We thus focused on DVL1 and hypothesized that RELA suppressed β‐catenin translocation to the nucleus through repressing the DVL1 transcriptional activation. Indeed, CUT&Tag‐qPCR analysis revealed that RELA binding sites overlap with the promoter region of *DVL*1 (Figure 6C), suggesting a potential regulatory relationship between RELA and DVL1 transcription. Additionally, ablation of DVL1 using sg*DVL1* did not alter total protein levels of β‐catenin (Figure 6D) but did decrease its amounts in the nucleus but not cytoplasm of MT‐PHHs‐sg*RELA* (Figure 6E). Genes associated with Wnt/β‐catenin such as *LEF1* and *TCF7* were also downregulated, accompanied by an increase in *APC* expression in MT‐PHHs‐sg*RELA* upon DVL1 ablation (Figure 6F). Moreover, other downstream target genes of the Wnt/β‐catenin signaling, such as *CCND1* and *MMP7* (Figure S9A, Supporting Information), along with cell stemness‐related genes like *ANPEP*, *CD24* and *CD44* were also repressed in MT‐PHHs‐sg*RELA*, upon DVL1 deletion (Figure S9B, Supporting Information). Furthermore, knockout of DVL1 also compromised the colony formation capacity (Figure 6G) and invasion activity (Figure 6H) of MT‐PHHs‐sg*RELA*,. These results show that RELA deters β‐catenin nucleus translocation by inhibiting DVL1 expression in HCC.

### 2.8 RELA Agonist Exerted Antitumor Effects and Anti‐Metastatic Potential in TP53^R249S^ Mutant Hepatocellular Carcinoma

**Figure 7:**
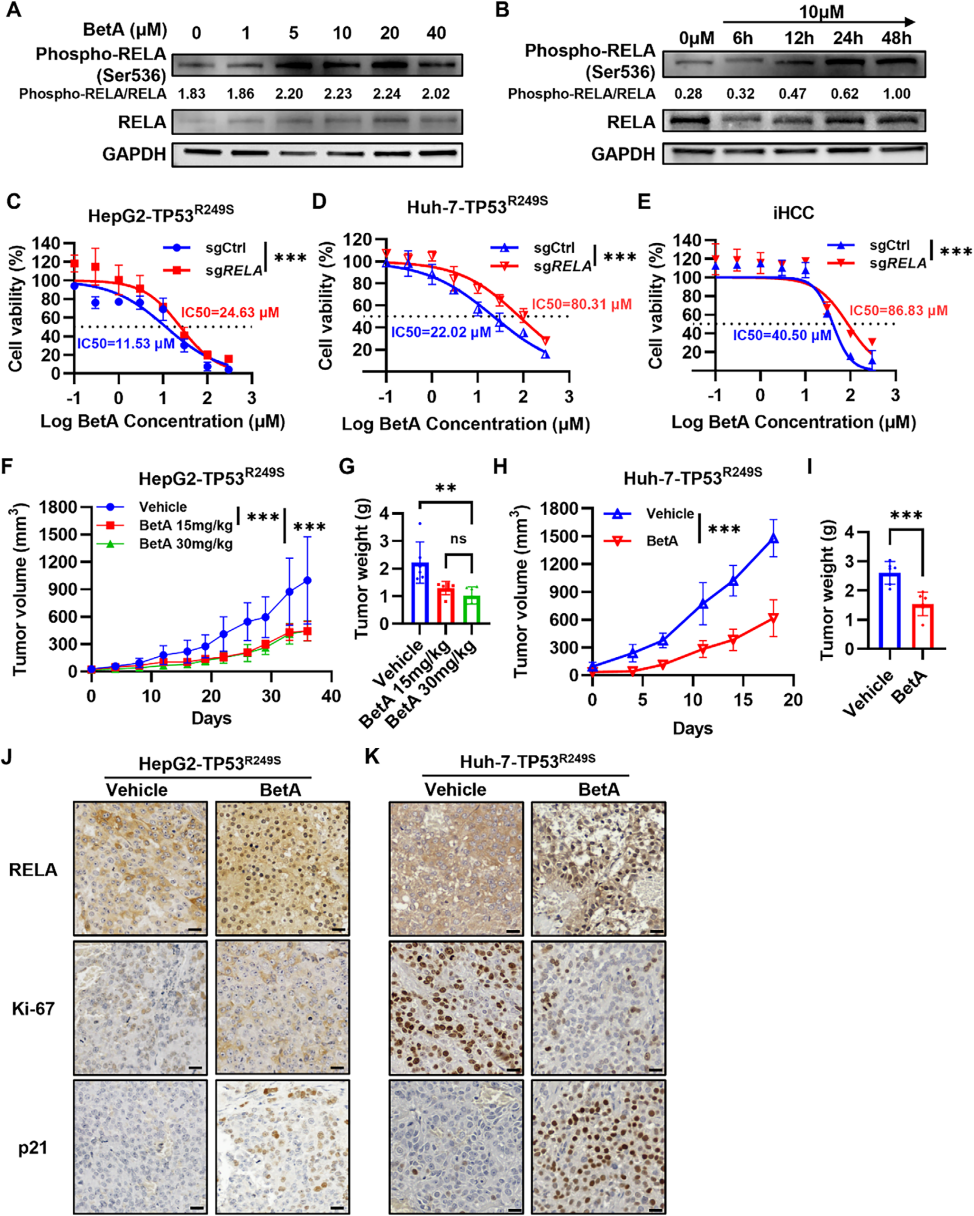
RELA agonist blocked tumorigenesis in TP53^R249S^ mutant hepatoma cells. A) Western blot analysis of the phosphorylation levels of RELA in HepG2‐TP53^R249S^ cells treated with 0, 1, 5, 10, 20 or 40 µM BetA for 24 h. The quantified phosphorylation levels of RELA are shown. Target protein signals were quantified by measuring the band intensities relative to GAPDH using ImageJ. B) Western blot analysis of the phosphorylation levels of RELA in HepG2‐TP53^R249S^ cells treated with 10 µM BetA for 6, 12, 24 or 48 h. The cells treated with 0 µM BetA were used as a negative control. The quantified phosphorylation levels of RELA are shown. C) IC50 of BetA in RELA‐deficient or RELA‐wildtype HepG2‐TP53^R249S^ cells. Error bars, mean ± SD. ***P ≤ 0.001, data were analyzed by two‐way ANOVA with Tukey's multiple comparison test. D) IC50 of BetA in RELA‐deficient or RELA‐wildtype Huh‐7‐TP53^R249S^ cells. Error bars, mean ± SD. ***P ≤ 0.001, data were analyzed by two‐way ANOVA with Tukey's multiple comparison test. E) IC50 of BetA in RELA‐deficient or RELA‐wildtype iHCC cells. Error bars, mean ± SD. ***P ≤ 0.001, data were analyzed by two‐way ANOVA with Tukey's multiple comparison test. F) Tumor growth curves of HepG2‐TP53^R249S^ xenograft mice treated with vehicle, 15 or 30 mg kg^−1^ BetA (*n* = 6). Vehicle or BetA were administered once every two days for 14 days (per oral). Error bars, mean ± SD. ***P ≤ 0.001, data were analyzed by two‐way ANOVA with Tukey's multiple comparison test. G) Tumor weight statistics are shown. Error bars, mean ± SD. **P ≤ 0.01, data were analyzed by two‐tailed unpaired Student's t‐test. H) Tumor growth curves of Huh‐7‐TP53^R249S^ xenograft mice treated with vehicle or 15 mg kg^−1^ BetA (*n* = 6). ***P ≤ 0.001, data were analyzed by two‐way ANOVA with Tukey's multiple comparison test. I) Tumor weight statistics are shown. Error bars, mean ± SD. ***P ≤ 0.001, data were analyzed by two‐tailed unpaired Student's t‐test. J) Representative images of IHC staining for nuclear RELA, Ki‐67 and p21 in tumors derived from vehicle and BetA (15 mg kg^−1^) treated HepG2‐TP53^R249S^ xenograft mice. Scale bar, 20 µm. K) Representative images of IHC staining for nuclear RELA, Ki‐67 and p21 in tumors derived from vehicle and BetA treated Huh‐7‐TP53^R249S^ xenograft mice. Scale bar, 20 µm.

Given that RELA acted as a tumor suppressor in HCC harboring the TP53^R249S^ mutation, we investigated whether betulinic acid (BetA), a known agonist that promotes RELA activation,^[^ ^27^ ^]^ exerted antitumor effects in this genetic context. Following BetA treatment, RELA phosphorylation, which signifies its activation,^[^ ^28^ ^]^ was increased in HepG2‐TP53^R249S^ cells (**Figure** **7**A,B). To investigate the therapeutic potential of BetA in HCC harboring the TP53^R249S^ mutation, we determined the IC50 values of BetA across multiple TP53^R249S^‐overexpressing cell lines, including HepG2, Huh‐7 and iHCC.^[^ ^7^ ^]^ Of note, RELA ablation led to an increase in the IC50 values of BetA in HepG2‐TP53^R249S^ (Figure 7C), Huh‐7‐TP53^R249S^ (Figure 7D) and iHCC cells (Figure 7E). Conversely, the presence of RELA rendered these TP53^R249S^‐mutant cell lines more sensitive to BetA‐induced cytotoxicty (Figure 7C–E), suggesting that BetA‐induced cytotoxicity in this context was mediated through RELA activation. In xenograft models, BetA treatment significantly reduced tumor growth in both HepG2‐TP53^R249S^ (Figure 7F,G) and Huh‐7‐TP53^R249S^ xenografts (Figure 7H,I) while maintaining tolerability, as evidenced by stable body weights (Figure S10A,B, Supporting Information) and absence of acute toxicity (Figure S10C–F, Supporting Information) or other adverse effects.^[^ ^29^ ^]^ Mechanistically, IHC analysis on tumor samples from these xenografts showed that the nuclear translocation of RELA and nuclear expression of p21 were significantly increased in tumors from the BetA‐treated mice compared to the control group, while Ki‐67 levels were decreased (Figure 7J,K). These results demonstrate that RELA agonist BetA inhibits the growth of hepatoma cell lines with TP53^R249S^ overexpression in vivo.

In addition, we also assessed the potential anti‐metastatic effects of BetA in a Huh‐7‐TP53^R249S^‐induced metastatic model. In these experiments, BetA treatment slowed tumor dissemination (Figure S11A,B, Supporting Information) and prolonged survival compared to the vehicle‐treated group (Figure S11C, Supporting Information). These findings suggest an anti‐metastatic potential of BetA in liver cancer cells harboring the TP53^R249S^ mutation.

Collectively, our findings highlight the potential of applying RELA agonists to improve treatment outcomes for HCC patients with the TP53^R249S^ mutation.

## 3 Discussion

Identifying the key molecular events underlying HCC progression is crucial for improving personalized treatment strategies for HCC, and enhancing patient outcomes in HCC management. In our previous work, we demonstrated that the co‐occurrence of MYC, TP53^R249S^ and KRAS^G12D^ is sufficient to transform PHHs into in situ HCC, with all of them being indispensable for this process.^[^ ^7^ ^]^ However, the concurrent presence of all these three genetic alterations in a single HCC case is infrequent, which presents significant challenges in translating our findings regarding their interactions into clinical applications. Given the limited clinical relevance of KRAS^G12D^ in HCC, we sought to identify alternative tumor‐suppressive mechanisms that could compensate for its absence, particularly in the context of TP53^R249S^, which is highly prevalent in HCC patients. In this study, we excluded KRAS^G12D^ and performed an unbiased genome‐wide CRISPRko screen in MT‐PHHs to discover bona fide TSGs implicated in HCC transformation, and screened out several candidates including well‐known TSGs *CSK* and *NF2*. Notably, our findings provide direct genetic evidence that RELA acts as a tumor suppressor in human HCC progression, whose loss may functionally substitute for KRAS^G12D^ in driving HCC progression. Additionally, we propose that MYC is dispensable during this process (Figure 3F), and highlight the relationship between RELA's tumor‐suppressive role and the TP53^R249S^ genetic background (Figure S7, Supporting Information). TP53 is recognized as one of the most frequent alterations in HCC patients,^[^ ^30^ ^]^ among which, cases with the R249S mutation account for more than 90% of HCC patients that harbor TP53 mutations in developing countries such as China.^[^ ^6^ ^]^ Thus, the correlation between RELA and TP53^R249S^ defined in this study provides a more specific and precise classification in subclasses of HCC.

TP53 plays an important role in HCC progression. Consequently, TP53‐associated pathways have emerged as appealing therapeutic targets. However, due to the lack of typical target characteristics, strategies directly targeting TP53 are challenging to explore.^[^ ^31^ ^]^ Although several synthetic polypeptides and small molecules have been explored for restoring active conformation of mutant TP53,^[^ ^32^ ^]^ other selective targets related to TP53 need to be investigated. In this study, we propose the potential for rational use of the RELA agonist BetA in personalized therapeutic strategies against liver cancer, particularly in TP53^R249S^‐mutated HepG2 and Huh‐7 tumors that benefited from BetA administration (Figure 7; Figure S11, Supporting Information). These observations suggested RELA could be a potential therapeutic target for HCC patients with this TP53^R249S^ mutation.

Mechanistically, we demonstrated that the loss of RELA induces DVL1 upregulation, leading to increased nuclear translocation of β‐catenin and thereby promotion of MT‐PHHs cell stemness via the Wnt/β‐catenin signaling pathway (Figure 5 and Figure 6). The canonical Wnt signaling pathway is reported to play an important role in stemness and metastasis in HCC.^[^ ^26^, ^33^ ^]^ Although NF‐κB and Wnt signaling pathways regulate different gene subsets and influence cellular activities through independent cascades, recent research suggests that these two pathways can also interact through physical mediators and downstream target genes, forming a complex network. There is evidence proving that Wnt/β‐catenin signaling negatively regulates the NF‐κB/RELA signaling pathway.^[^ ^13^, ^34^ ^]^ Similarly, RELA has been demonstrated to directly interact with β‐catenin and suppress endothelial Wnt/β‐catenin signaling, resulting in acute neuroinflammation.^[^ ^35^ ^]^ NF‐κB can also trigger β‐catenin degradation through Smurf1 and Smurf2, inhibiting the osteogenic differentiation of mesenchymal stem cells (MSCs).^[^ ^36^ ^]^ These findings imply that Wnt/β‐catenin and NF‐κB/RELA could exert opposite cellular functions within the same cell, affecting a dynamic balance that ultimately influences overall cell behavior. Our observations support the hypothesis that RELA negatively regulates Wnt/β‐catenin signaling via direct suppression of DVL1 (Figure 6); however, the mechanism by which RELA dysregulates DVL1 remains unclear. Previous studies have established that transcriptional factors can recruit corepressors like histone deacetylases (HDACs) to switch from an activator to a repressor in specific contexts.^[^ ^37^, ^38^ ^]^ Alternatively, RELA may compete with activating transcription factors for binding to the DVL1 promoter, thereby suppressing its transcription, as previously reported for NF‐κB family members.^[^ ^39^ ^]^ To clarify the precise mechanisms underlying RELA‐mediated DVL1 suppression, further investigations are required.

Collectively, our findings provide evidence for a TP53^R249S^‐dependent role for RELA in suppressing HCC development. Previous studies revealed that RELA acts as an oncogene in several cancers, such as lung cancer,^[^ ^40^ ^]^ breast cancer^[^ ^41^ ^]^ and pancreatic cancer.^[^ ^42^ ^]^ Some studies have also confirmed the tumor‐promoting function of RELA in HCC.^[^ ^43^ ^]^ However, these studies haven't considered RELA's role in combination with other frequent genetic alterations in HCC, such as TP53 and MYC. While utilizing a humanized spontaneous HCC mouse model to assess RELA's role alongside TP53^R249S^ and MYC, surprisingly, we found that only TP53^R249S^ and RELA deficiency were dispensable to this process (Figure 3). However, as our study was limited to hepatomas with TP53^R249S^ overexpression, further evidence is needed to explore whether the tumor‐suppressive effects of RELA are dependent on this specific TP53^R249S^ mutation or extend to other TP53 dysfunction mutations as well. Indeed, a disrupted metabolic stabilization, which could be driven by TP53 dysfunction mutations,^[^ ^44^ ^]^ has been reported to shift hepatic NF‐κB/TNF signaling from a pro‐survival to a pro‐apoptotic pathway, eventually leading to hepatocyte deterioration.^[^ ^45^ ^]^ Thus, we postulate that RELA's tumor‐suppressive role may not be limited to the TP53^R249S^ mutation alone.

Clinically, our data suggest that RELA dysfunction occurs in a substantial proportion of HCC cases (Figure S3D,E, Supporting Information), with low RELA expression showing a significant association with larger tumor size (Table 1). These results suggest RELA's potential role in restraining HCC growth, such as regulating cell cycle‐related pathways. Strikingly, in the subgroup of low‐grade or early‐stage HCC patients, reduced RELA expression positively correlates with poorer prognosis (Figure 2A,B). This stage‐specific prognostic pattern implies RELA dysfunction is an early molecular event in hepatocarcinogenesis. Notably, TP53 alteration has also been identified as an early event for HCC progression.^[^ ^5^ ^]^ To this end, we speculate that the observed phenotypes, such as enhanced cell proliferation and promoted EMT driven by RELA loss in TP53^R249S^‐overexpressing cells, might be dependent on the specific premalignant context of the cells used in this study. Similar to RELA, other genes such as p53 and FOXA1 were also shown to have diverse roles in liver diseases.^[^ ^46^ ^]^ This phenomenon might stem from liver's distinct regenerative biology, which reverses conventional oncogene/tumor suppressor paradigms observed in other tissues. These stage‐specific functional switches underscore the critical need for stage‐tailored therapeutic strategies.

In conclusion, our findings demonstrate that RELA is an essential suppressor of HCC progression, and underscore the correlation between RELA's tumor‐suppressive role and the TP53^R249S^ mutation. Moreover, activation of RELA by agonist BetA effectively suppresses tumor growth of HepG2 as well as Huh‐7 cells with this TP53^R249S^ mutation, highlighting BetA's potential as a therapeutic strategy for HCC, particularly in subtypes harboring TP53 mutations.

## 4 Experimental Section

### Genome‐Wide CRISPR‐Cas9 Screen

The human CRISPR KO library (GeCKO v2) and lentiCas9‐Blast plasmid for genome‐wide CRISPR‐Cas9 screen were obtained from Addgene (#1 000 000 049). Amplification of the library and preparation of the lentivirus were performed following the protocol provided by Addgene with *E. coli* DH5α Electro‐cells (TaKaRa, #9027). To ensure the diversity of the GeCKO v2 library (GeCKOa), which contains 65383 sgRNAs, a minimum of 60 million MT‐PHH cells stably expressing Cas9 were needed for infection with the gRNA lentivirus library. The infection was carried out at a low multiplicity of infection (MOI) (≈0.3), aiming for an average 300‐fold coverage of each sgRNA. After selection by puromycin (2 µg mL^−1^, Sigma) for 2 days, the mutant cell pool was cultured for another 7 days. To perform the genome‐wide screens, 6 × 10^6^ cells were subcutaneously transplanted into individual NSI (NOD/SCID/IL2rγ^−/−^) animals for each screen, and 3 × 10^7^ transduced cells were harvested as reference samples (Inputs). After the screens, total genomic DNA of cells or tumors was extracted using StarSpin Universal DNA Kit (GenStar, Beijing, China). SgRNA PCR amplification was conducted according to Zhang's protocols.^[^ ^47^ ^]^ Subsequently, high‐throughput sequencing of the PCR products was performed by Novogene. SgRNA read counts were quantified and analyzed using MAGeCK v0.5.7 software,^[^ ^16^ ^]^ with 1000 non‐targeting sgRNAs included in the sgRNA library serving as negative controls. Positive hits were defined as sgRNAs demonstrating a significant skew (≥ 10‐fold change versus non‐targeting controls) in representation compared to these built‐in non‐targeting sgRNAs. To ensure the robustness of the screen, the final list of selected sgRNA‐targeted genes was required to include known HCC dependency genes, thereby validating the screening results.

### Cells and Culture Conditions

Primary human hepatocytes (PHHs) were rethawed at 37 °C in thaw medium (Cat#LV‐Rec001/3), and recovered in maintenance medium (Cat#LV‐WEM001) in coated culture dishes precoated with Type I collagen (Cat#LV‐Collagen001). MT‐PHHs were obtained from PHHs with introduction of pWPXLD‐MYC‐eGFP‐luciferase and pWPXLD‐TP53^R249S^‐eGFP‐luciferase, and cultured in Dulbecco's Modified Eagle Medium/Nutrient Mixture F‐12 (DMEM/F12) (Gibco, Grand Island, NY, USA), supplemented with 10% heat‐inactivated FBS (Gibco, Grand Island, NY, USA) and 1% penicillin/streptomycin (Gibco, Grand Island, NY, USA). For the 3D culture, cells were trypsinized and resuspended at a concentration of 10 000 cells mL^−1^ and added to a solution of 50% Matrigel (Corning, Cat#356234) containing culture medium. Then 100 µL of cells were plated in 96‐well plates (Thermo Fisher Scientific) coated with Matrigel and grown for up to 6 days. All cells were cultured at 37 °C in an atmosphere of 5% CO_2_.

### Lentivirus Production

SgRNAs targeting *CSK*, *NF2*, *RELA*, *SGK3*, *DPAGT1*, *DCBLD2*, *TOE1*, *LIMD1*, or *DVL1* were cloned into LentiCRISPR v2 vectors (Addgene, #52961). ShRNAs targeting CTNNB1 were cloned into pLKO.1 vectors. The sequences of sgRNAs or shRNAs were listed in Table S2 (Supporting Information).

Lentivirus particles were produced in HEK‐293T cells following polyethyleneimine (PEI) (4955393‐7, Polysciences, Inc., USA)‐mediated transfection. To produce lentivirus, lentiviral vectors were co‐transfected with psPAX2 and pMD2.G (Addgene). After 10 h of transfection, medium was changed and cell incubation continued. At 48–72 h post‐transfection, supernatant containing the viral particles was collected and filtered through a 0.45 µm filter to remove any cell debris. If necessary, the virus was concentrated using ultracentrifugation. The viral stock was stocked at −80 °C for future use. All procedures were performed under a biosafety hood to maintain safety throughout the experiment.

### Western Blot Assays

Western blotting was performed following a routine protocol. The cells were lysed in RIPA buffer (Pierce, Rockford, Illinois, USA) and proteins were quantified using a BCA Protein Assay kit (Pierce, Rockford, IL, USA). An equal amount of total protein lysate was separated by 5 × SDS–PAGE and transferred to a PVDF membrane (Millipore). The membrane was blocked with 5% (w/v) Bovine Serum Albumin (BSA) and incubated overnight with primary antibodies at 4 °C followed by HRP‐conjugated secondary antibody (Cell Signaling Technology, Boston, USA) incubation for 1 h. The protein bands were visualized using an enhanced chemiluminescence kit (ECL Plus, General Electric Healthcare, Little Charfont, UK). The antibodies were listed in Table S2 (Supporting Information).

### Cell Proliferation Assays

To evaluate cell proliferation, cells were seeded into 6‐well plates at an initial density of 20 000 cells per well in 2 mL of complete DMEM/F12. The cells were allowed to adhere overnight at 37 °C in a humidified atmosphere of 5% CO_2_. Cells were incubated under standard culture conditions and harvested at specific time points (24, 48, and 72 h). The proliferation rate was calculated based on the increase in cell number over time. Each experiment was performed in triplicate to ensure accuracy and reproducibility.

### Long‐Term Cell Proliferation Assays (Colony Formation Assays)

To evaluate the long‐term proliferative capacity of cells, cells were seeded into 6‐well plates (10 000 cells per well) and were incubated at 37 °C in a humidified atmosphere with 5% CO_2_ for 10–14 days. The medium was replaced twice weekly. After the incubation period, when the colonies were formed, cells were gently washed and fixed with 4% paraformaldehyde solution and stained with 0.1% crystal violet solution. The numbers of colonies containing 50 or more cells were counted using an inverted microscope. Each experiment was performed in triplicate to ensure accuracy and reproducibility.

### Soft‐Agar Assays

The base layer was prepared by mixing 1.0% agarose with pre‐warmed complete 2×DMEM/F12 at a 1:1 ratio, resulting a final agar concentration of 0.5%. For the top layer, cells were suspended in complete DMEM/F12 containing 0.7% agarose at a density of 5000–10 000 cells per well. The cell suspension was then carefully layered over the solidified base agar in the 6‐well plates. After the top layer had solidified, 2 mL of complete DMEM/F12 was added to each well to prevent drying. The plates were incubated at 37 °C in an atmosphere of 5% CO_2_ for 10–14 days. Every 3 days, 1 mL of fresh complete DMEM/F12 was gently added to each well. At the end of the incubation period, the number and size of the colonies were then quantified using a dissecting microscope.

### CUT&Tag‐qPCR Analysis

The CUT&Tag (Cleavage Under Targets and Tagmentation) assays was performed according to the protocol described by Kaya‐Okur et al.^[^ ^48^ ^]^ Briefly, MT‐PHH cells were harvested and washed with Wash Buffer (20 mM HEPES pH 7.5, 150 mM NaCl, 0.5 mM Spermidine, 1× protease inhibitor). Cells were bound to Concanavalin A‐coated magnetic beads and permeabilized with Digitonin‐containing buffer (0.01%–0.05%). Target‐specific primary antibodies against RELA (Cell Signaling Technology, D14E12; dilution 1:100) were incubated overnight at 4 °C, followed by incubation with a secondary antibody. The protein A‐Tn5 transposase complex (pre‐loaded with sequencing adapters) was then added to perform tagmentation at 37 °C for 1 h. DNA was extracted using StarSpin Universal DNA Kit (GenStar, Beijing, China), and qPCR amplification was performed using *PerfectStart* Green qPCR SuperMix (TransGene, Beijing, China) with primers specific to the genomic regions of interest (primer sequences listed in Table S3, Supporting Information).

### Quantitative Real‐Time PCR

mRNA was extracted using StarSpin Small RNA Kit (GenStar, Beijing, China) and reverse transcribed into cDNA using the TransScript II All‐in‐One First‐Strand cDNA Synthesis SuperMix for qPCR (One‐Step gDNA Removal) (TransGene, Beijing, China). All reactions were performed with TransScript II Probe One‐Step qRT‐PCR SuperMix (TransGene, Beijing, China) on a Bio‐Rad CFX96 real‐time PCR machine (Bio‐Rad, Hercules, CA). The qRT–PCR primers are listed in Table S3 (Supporting Information). Delta CT calculations were relative to β‐actin and corrected for PCR efficiencies.

### Histological Assays

Tissue samples were fixed with 4% formalin, embedded in paraffin, sectioned at a thickness of 4 µm, and stained with H&E. Images were acquired with a microscope (Leica DMI6000B, Leica Microsystems, Wetzlar, Germany). Paraffin sections were used for immunohistochemical (IHC) staining. A primary antibody (1:100–1:2000) was added to the tissue sections on slides and incubated overnight at 4 °C. Alexa Fluor 647‐conjugated goat anti‐mouse (1:2000) and Alexa Fluor 568/488‐conjugated goat anti‐rabbit (1:2000) antibodies were added and incubated for 1 h at room temperature. DAPI was used to stain nuclei, and the prepared slides were analyzed using a Zeiss LSM800 microscope. Dilutions were prepared according to the product information. The antibodies are listed in Table S2 (Supporting Information).

### Animal Experiments

All animal experiments were performed in the Laboratory Animal Center of GIBH and were approved by the GIBH Animal Welfare Committee (Approval No.: A5748‐01). All protocols were approved by the relevant Institutional Animal Care and Use Committee (IACUC). The study utilized 6‐ to 8‐week‐old NSI or NSIF mice, weighing ≈18–22 g, with two strains of immunodeficient mice bred and maintained in specific pathogen‐free (SPF)‐grade facilities. Mice were housed in individually ventilated cages with controlled temperature and humidity and were provided autoclaved food and water ad libitum. Both male and female mice were included and were randomized into experimental groups consisting of at least five animals per group. All animals were free of known pathogens and had no prior experimental procedures before inclusion in this study.

For the in vivo xenograft experiment, tumor cells in 120 µL PBS were subcutaneously injected to establish subcutaneous (flank) tumors. During the experimental period, tumor measurements were taken every 3 days using a caliper. Tumor volumes were calculated using the following equation: length (a) × width (b)^2^)/2. After the tumors in the control group reached 1000 mm^3^, the mice were sacrificed, and the final tumor weights were determined; the selected tissues were then subjected for further analysis.

For the in vivo assay of the RELA agonist, betulinic acid (BetA) was dissolved in vehicle (5% v/v DMSO in olive oil). For evaluating the antitumor effects of BetA, subcutaneous xenograft models were established by implanting HepG2‐TP53^R249S^ or Huh‐7‐TP53^R249S^ into mice, which were randomly assigned to control and treatment groups when the tumor volume reached 50 mm^3^. For orthotopic assessment, tumor cells were intrasplenically injected, and tumor progression was monitored via live imaging. To investigate BetA's anti‐metastatic potential, peritoneal metastasis was induced by intraperitoneal injection, with metastatic spread tracked longitudinally by imaging. BetA (15 or 30 mg kg^−1^) was administered orally every two days for 2 weeks. BetA doses used in this study were selected on the basis of previous studies.^[^ ^49^ ^]^

To evaluate the acute toxicity of BetA, male and female NSI mice (*n* = 5 per group) were administered BetA at 5× and 10× the experimental therapeutic dose (75 and 150 mg kg^−1^, respectively) via oral gavage. Body weights and clinical signs (mortality, lethargy, abnormal behavior) were monitored daily for 14 days. At the endpoint, mice were euthanized, and key organs (kidneys, lungs, spleens, livers and ovaries or testes) were harvested for histopathological analysis. Tissues were fixed in 4% paraformaldehyde, embedded in paraffin, sectioned, and stained with hematoxylin and eosin (H&E) to assess potential toxicity‐induced morphological changes.

For the establishment of long‐term humanized spontaneous orthotopic models using NSIF mice, primary human hepatocytes were incubated with the cocktail of lentivirus 24 h after cell recovery. Then, 0.5–1 million PHHs were transplanted into the spleens of every NSIF mice after the withdrawal of NTBC‐containing drinking water.^[^ ^50^ ^]^ The NTBC concentration was gradually decreased (3.5 mg L^−1^, days 0 to 2; 1.75 mg L^−1^, days 3 and 4; and 0.8 mg L^−1^, days 5 and 6), and NTBC was then completely withdrawn 1 week after transplantation. NTBC was readministered with gradient concentration for 5 days once the transplanted mouse had lost > 20% of its pretransplant weight. The dying recipient mice were euthanized to collect liver samples.

### Bulk RNA Sequencing

mRNA extracted from MT‐PHHs transduced with sgCtrl or sg*RELA* was prepared according to the TruSeqTM RNA Sample Preparation Guide, and sequencing was performed on a BGISEQ‐500 (BGI, Wuhan, China). Sequencing reads were mapped to the human RefSeq‐RNA reference sequence using the FANSe2 algorithm. Reads mapped with tophat2 were associated with genes using the custom Perl scripts that allowed no more than 2 unmapped bases. Cufflinks (version 2.1.1) was used to identify reads consistent with the annotated genes. These genes were quantified using the reads per kilobase million (RPKB) method. For small genes (≤ 200 bps), a minimum of 10 mappable reads were required. Mappable reads were imported into the DEGseq software package to calculate up‐ or down‐regulation of genes.

### Human Samples

All HCC patient samples were collected for clinical studies with detailed demographic and clinical characteristics provided in Table S1 (Supporting Information). Tumor and adjacent non‐tumor tissues were obtained during surgical procedures at the LKS Faculty of Medicine, The University of Hong Kong, with written informed consent compliance from all participants. The samples were immediately snap‐frozen in liquid nitrogen and stored at −80 °C until further analysis. The study protocol was performed in accordance with the provisions of the Helsinki Declaration of 1975 and was approved by the Institutional Review Board of the University of Hong Kong (IRB approval no.: UW_11–099). Written informed consent was obtained from all subjects.

### Statistical Analysis

All data were presented as the mean ± SD values, and significances were evaluated by Student's t‐test (two groups) or ANOVA with Tukey's multiple comparison test (three or more groups) as indicated. *N* indicates the number of animals per group as specified in figure legends.

All statistical tests were chosen after verifying assumptions of normality (Shapiro‐Wilk test) and equal variance (Brown‐Forsythe test). For analysis of IC50 determinations, compound concentrations were log10‐transformed to linearize the concentration‐response relationship. The transformed data were then fitted using nonlinear regression to calculate half‐maximal inhibitory concentrations.

Kaplan–Meier analysis of the correlation between candidate genes expression and overall survival was acquired from the Kaplan‐Meier Plotter (https://kmplot.com/analysis/), which was based on datasets from The Cancer Genome Atlas (TCGA). Survival was plotted using a Kaplan‐Meier survival curve, and statistical significance was determined by the log‐rank (Mantel‐Cox) test.

All statistical analyses were performed using Prism version 7.0 (GraphPad, Inc., San Diego, CA, USA). *P ≤ 0.05; **P ≤ 0.01; ***P ≤ 0.001; ns: not significant.

## Conflict of Interest

The authors declare no conflict of interest.

## Author Contributions

Z.Wu, Z.Wang and D.Z. contributed equally to this work. Z.Wu, D.Z. designed and performed the experiments and analyzed the data. Z.Wu and D.Z. performed the bioinformatical analyses of the RNA‐seq. K.M. provided clinical samples of human HCC. Z.Wang and Y.Z analyzed clinical and TCGA data. Z.Wu performed in vivo experiments and analyzed data. Y.Z., H.J., Z.D., L.J., Q.W., Y.L., S.L., and Y.Y. participated in preparing and performing experiments. P.L., K.M., J.P. and Z.J gave suggestions. P.L. supervised the project. P.L., Z.Wu, D.Z., J.L. and G.C. wrote and/or revised the manuscript. All authors discussed and commented on the manuscript.

## Ethics Statements

All animal experiments were approved by the IACUC of the Guangzhou Institute of Biomedicine and Health (Approval No.: A5748‐01) and conducted in accordance with institutional guidelines. Human subject research was approved by the Institutional Review Board of the University of Hong Kong (IRB approval No.: UW_11‐099), and written informed consent was obtained from all participating individuals.

## Supporting information



Supporting Information

Supporting Data

## Data Availability

The data that support the findings of this study are available from the corresponding author upon reasonable request.
