# Supplementary material for: RELA Ablation Contributes to Progression of Hepatocellular Carcinoma with TP53R249S Mutation and is a Potential Therapeutic Target
Source: Adv Sci (Weinh). 2025 Sep 8;12(48):e00335. doi: 10.1002/advs.202500335 (PMC12752641; doi:10.1002/advs.202500335)
Supplement: Supplementary file 1 — Supporting Information [file ADVS-12-e00335-s001.docx]

Supporting Information

**RELA Ablation Contributes to Progression of Hepatocellular Carcinoma with TP53^R249S^ Mutation and Is a Potential Therapeutic Target**

*Zhiping Wu, Zhe Wang, Diwei Zheng, Yongfang Zheng, Zhiwu Jiang, Jiang Lv, Yueqin Zhu, Heng Jia, Ziyuan Duan, Tingjie Yuan, Qiting Wu, Youguo Long, Shouheng Lin, Yao Yao, Georgia Carson, Jean P. Thiery, Kwan Man*, Peng Li**

**This PDF file includes:**

**Figure S1 to S11**

**Table S1 to S3**

**Supplementary Figures**

**
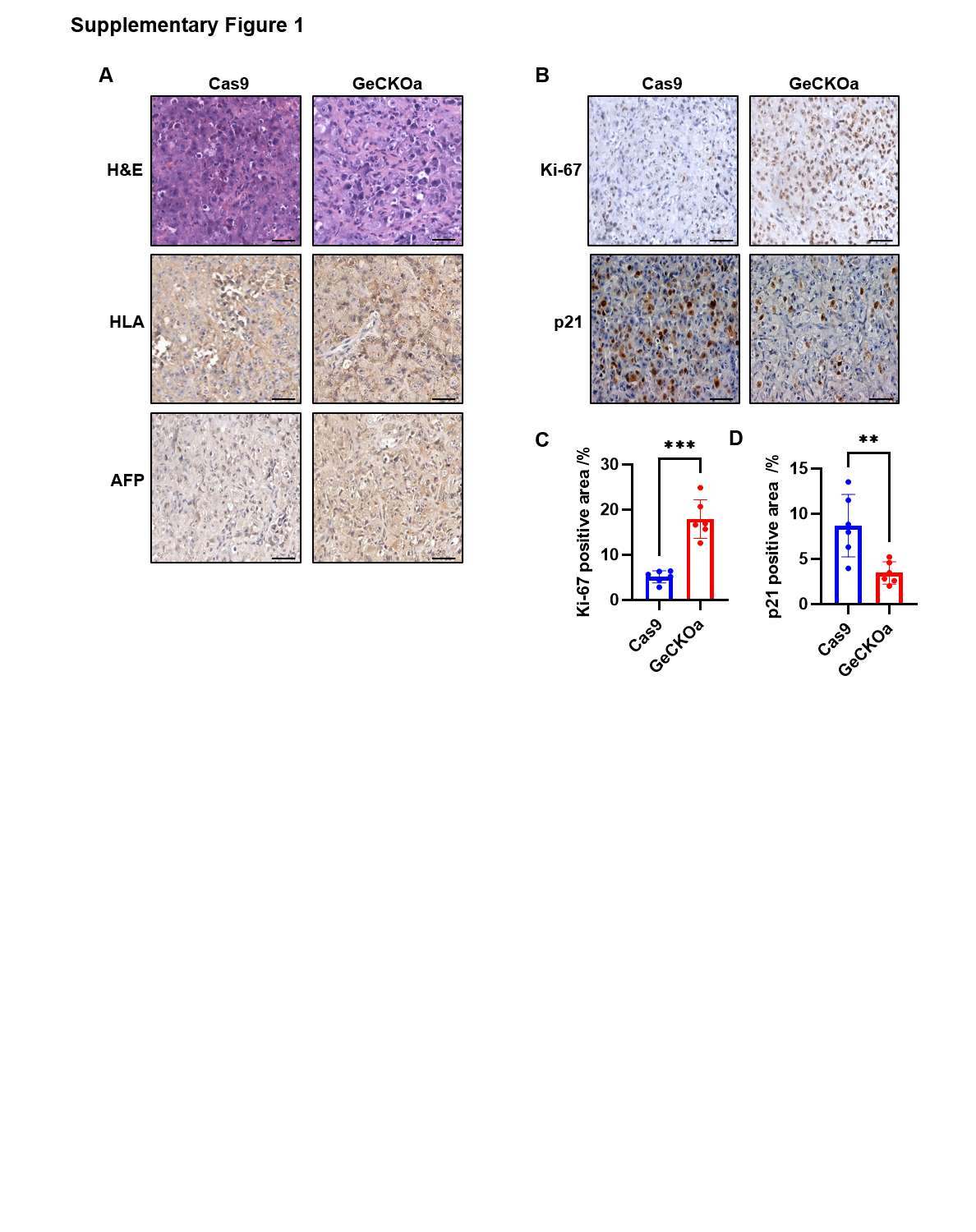
**

**Figure S1. Histological analysis of tumors demonstrating HCC advancement in GeCKOa Group.**

(A) Representative images of H&E staining and IHC staining for HLA and AFP in tumor tissues derived from Cas9 and GeCKOa Group. Scale bars, 50 μm. (B) Representative images of IHC staining for Ki-67 and p21 in tumor tissue derived from Cas9 and GeCKOa Group. Scale bars, 50 μm. (C) Quantification of Ki-67^+^ areas in Fig. S1B. Error bars, mean ± SD. ****P* ≤ 0.001, data were analyzed by two-tailed unpaired Student’s t-test. (D) Quantification of p21^+^ areas in Fig. S1B. Error bars, mean ± SD. ***P* ≤ 0.01, data were analyzed by two-tailed unpaired Student’s t-test.


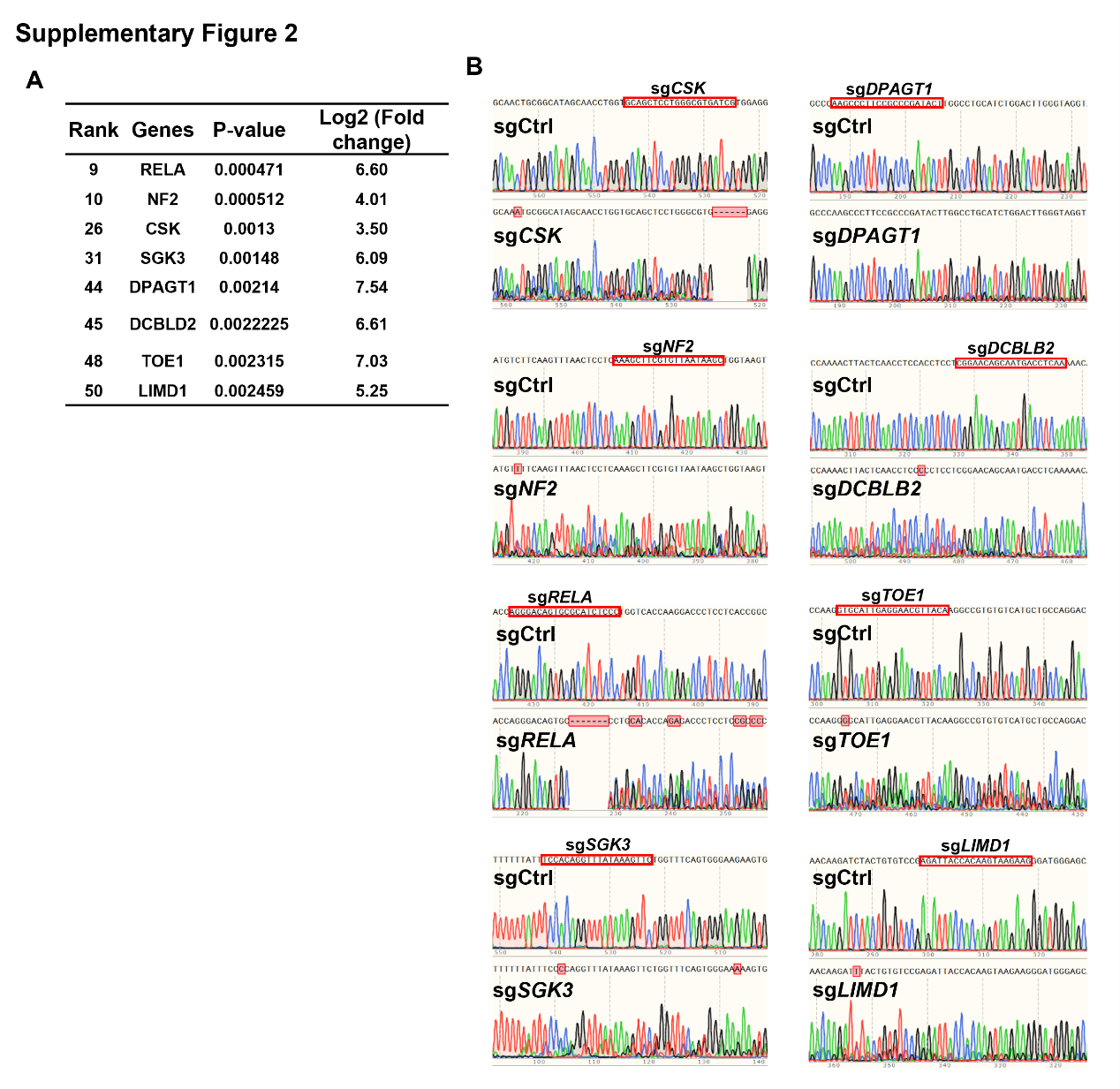


**Figure S2. Sanger sequencing results from secondary validation.**

(A) Table summarizing candidate genes identified by MAGeCK analysis and robust rank aggregation (RRA) ranking of top depleted genes in GeCKOa-transduced MT-PHHs derived transformed tumors. (B) Sanger sequencing of the genomic DNA (gDNA) of MT-PHHs transduced with sgRNAs targeting *CSK*, *NF2*, *RELA*, *SGK3*, *DPAGT1*, *DCBLD2*, *TOE1* and *LIMD1*. The double peaks and base deletion confirm the activity of sgRNAs.


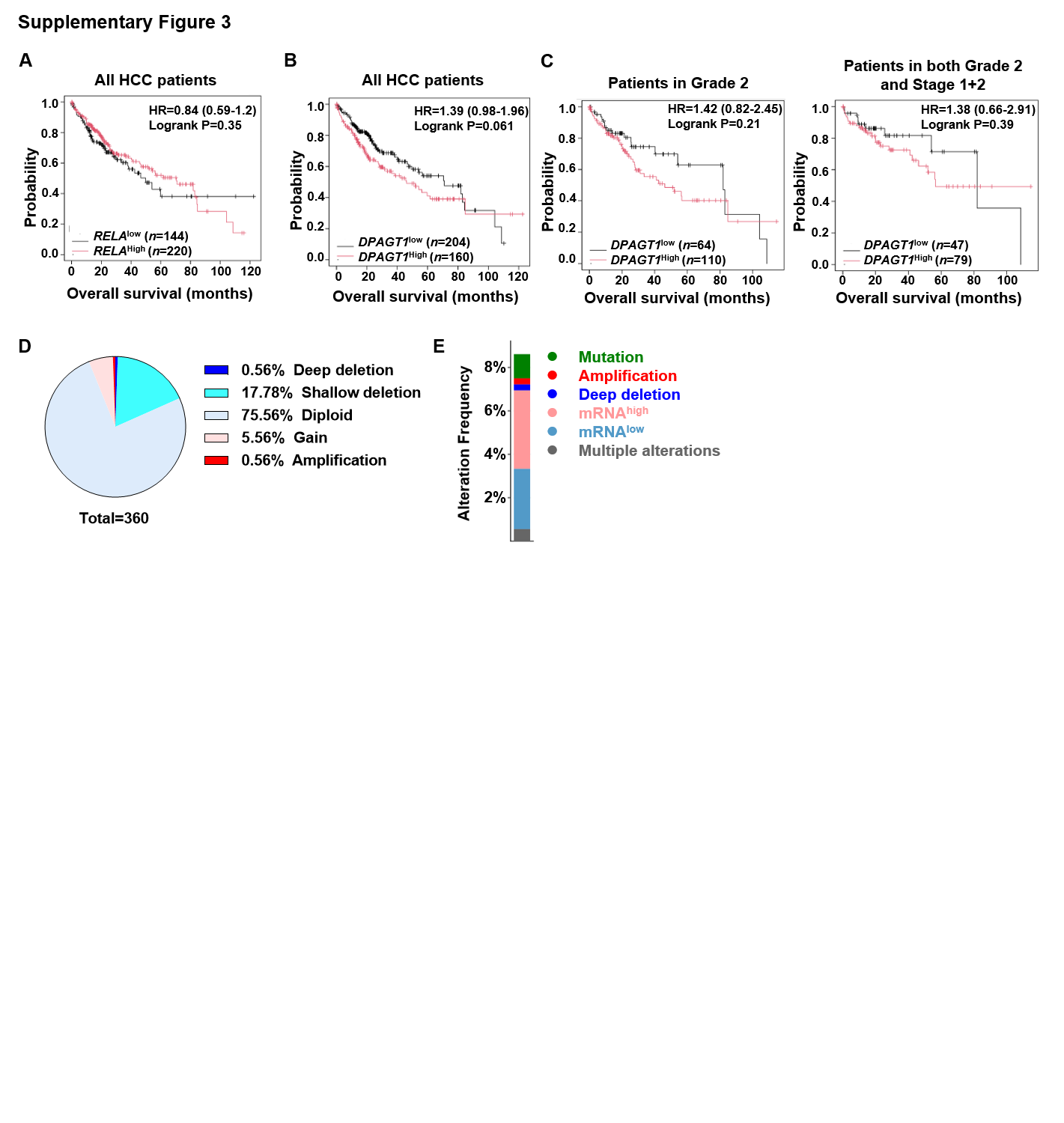


**Figure S3. Analysis of the TCGA-LIHC database.**

(A) Kaplan–Meier analysis of the overall survival of patients with low (*n* = 143) or high (*n* = 221) *RELA* expression in the TCGA-LIHC database. (B) Kaplan–Meier analysis of the overall survival of patients with low (*n* = 204) or high (*n* = 160) DPAGT1 expression in the TCGA-LIHC database. (C) Kaplan–Meier analysis of the overall survival of TCGA HCC patients in Grade 2 with low (*n* = 64) or high (*n* = 110) RELA expression and in both Grade 2 and Stage 1+2 with low (*n* = 47) or high (*n* = 79) RELA expression. (D) The distributions of *RELA* copy number variations, including deep deletion, shallow deletion, diploid, gain and amplification in the TCGA-LIHC database. (E) The percentages of genomic alteration types, including mutation, amplification, deep deletion, mRNA^low^, mRNA^high^ and multiple alterations in the TCGA-LIHC database.

**
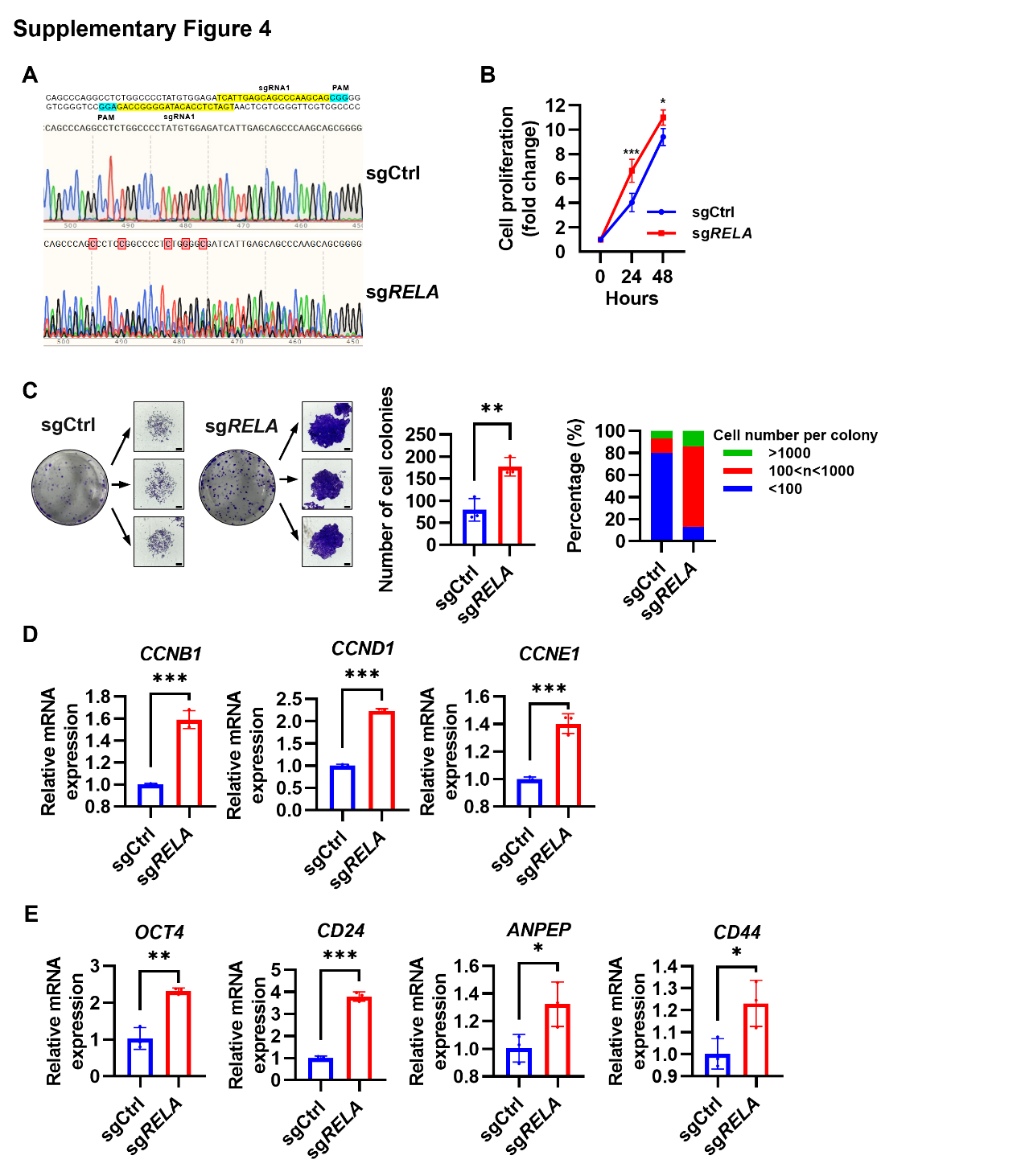
**

**Figure S4. RELA deficiency enhanced cell growth and promoted stemness in MT-PHHs.**

(A) Sanger sequencing of gDNA of cells transduced with a pair of sgRNAs targeting *RELA*. The double peaks and base aberration confirm the activity of sgRNAs. (B) Representative proliferation curves for MT-PHHs-sgCtrl and MT-PHHs-sg*RELA*. Error bars, mean ± SD. **P* ≤ 0.05; ****P* ≤ 0.001, data were analyzed by two-way ANOVA with Tukey's multiple comparison test. (C) Representative images of colonies formed by MT-PHHs-sgCtrl or MT-PHHs-sg*RELA*, stained with crystal violet. Scale bars, 200 μm. The quantification of colony number and size are shown on the right. Error bars, mean ± SD. ***P* ≤ 0.01, data were analyzed by two-tailed paired Student’s t-test. The colony size distributed is distinguished by ≤ 100 cells, 100 ≤ n ≤ 1000 cells, and > 1000 cells. (D) Relative mRNA levels of *CCNB1*, *CCND1*, *CCNE1* in MT-PHHs-sgCtrl and MT-PHHs-sg*RELA*, based on quantitative RT-PCR. Error bars, mean ± SD. ****P* ≤ 0.001, data were analyzed by two-tailed paired Student’s t-test. (E) Relative mRNA levels of *OCT4*, *CD24*, *ANPEP*, *CD44* in MT-PHHs-sgCtrl and MT-PHHs-sg*RELA*, based on quantitative RT-PCR. Error bars, mean ± SD. **P* ≤ 0.05, ***P* ≤ 0.01, ****P* ≤ 0.001, data were analyzed by two-tailed paired Student’s t-test.


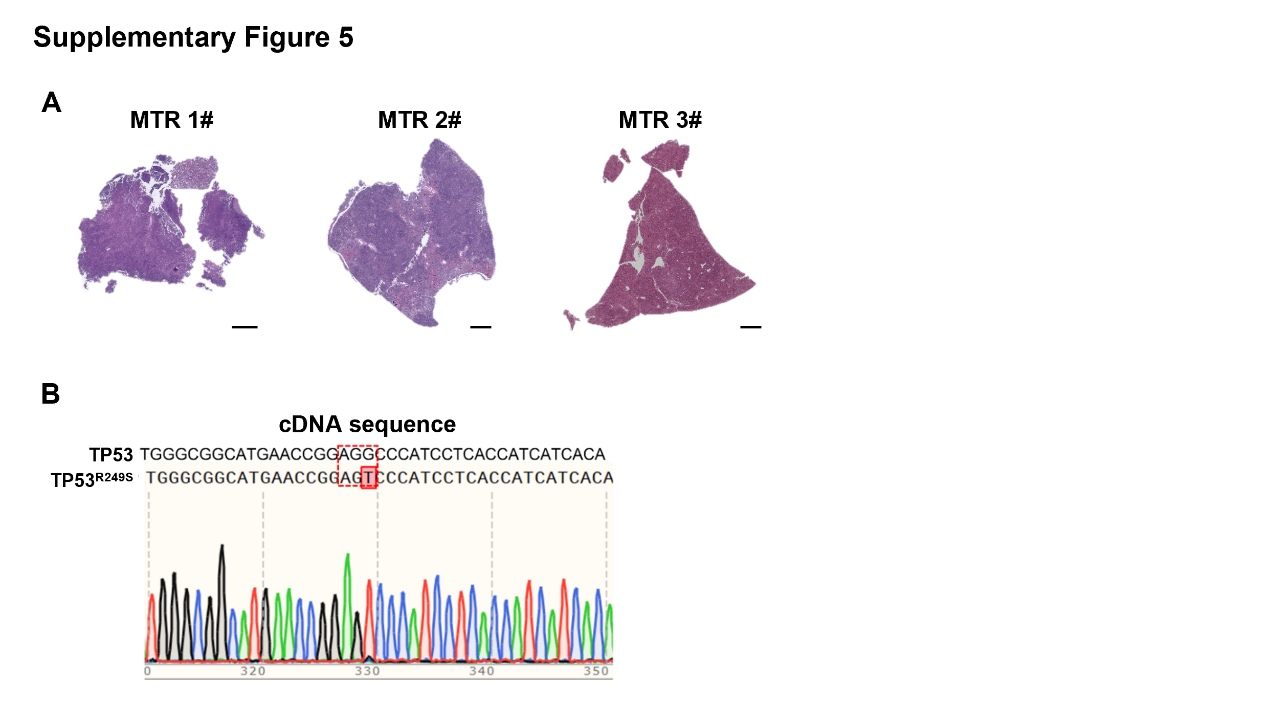


**Figure S5. Histological morphology of NSIF mice receiving MTR-transduced PHHs.**

(A) Representative H&E staining of the remaining liver tissues from NSIF mice receiving MTR-transduced PHHs. Scale bar, 1000 μm. (B) Sanger sequencing of the complementary DNA (cDNA) isolated from tumor tissues, the red dotted box indicates the R249S mutation in TP53, confirming the translation of TP53^R249S^.

**
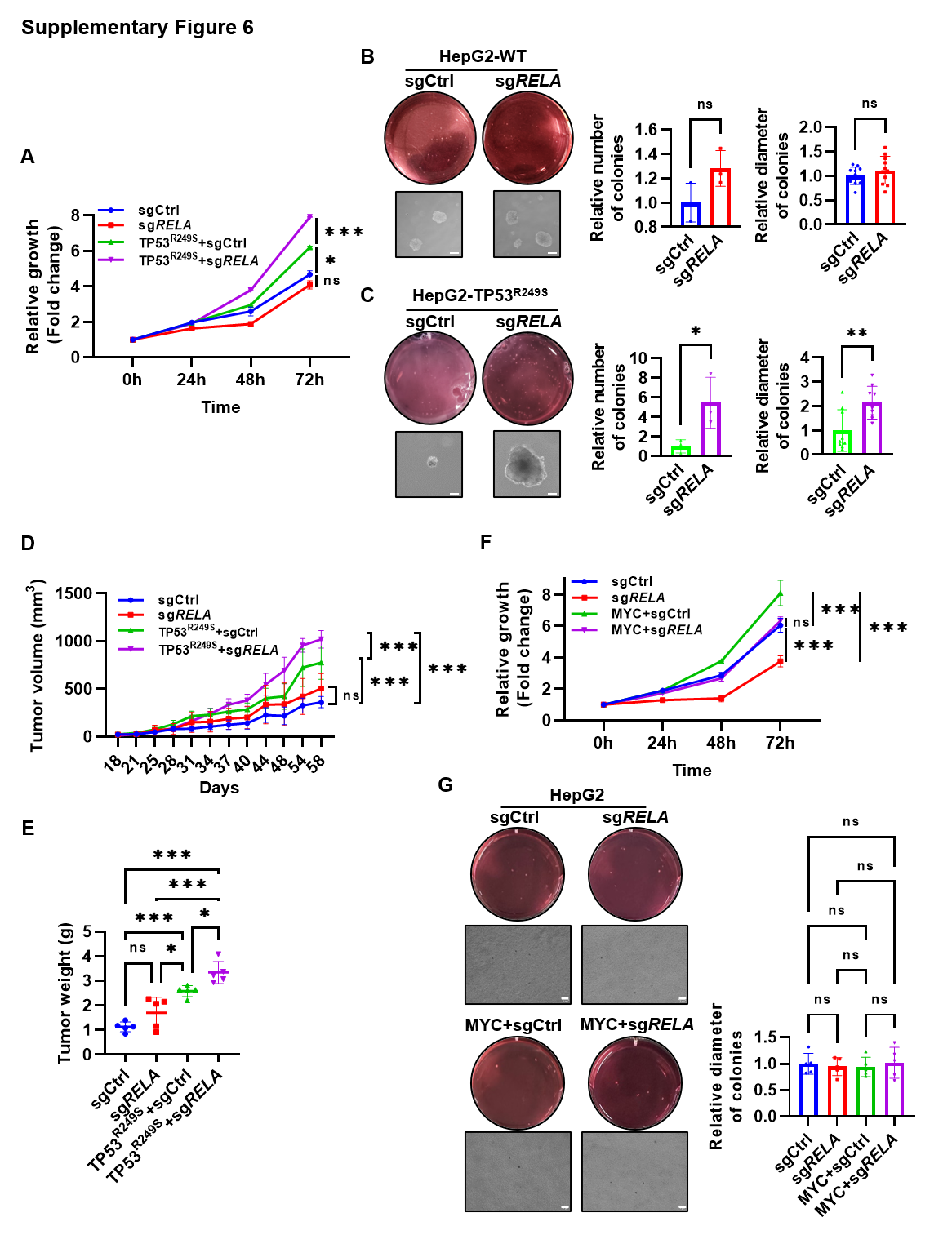
**

**Figure S6. RELA acted as a tumor suppressor in a TP53^R249S^ mutation-dependent manner.**

(A) Representative proliferation curves for HepG2-WT or HepG2-TP53^R249S^ transduced with sg*RELA* and sgCtrl respectively. Error bars, mean ± SD. **P* ≤ 0.05; ****P* ≤ 0.001, data were analyzed by two-way ANOVA with Tukey's multiple comparison test. (B) Soft agar colony formation assay of sgCtrl- and sg*RELA*-transduced HepG2-WT cells. The images are representative of three independent experiments. Full-view images show the number of colonies, and the 4× magnification images indicated the size of the colonies. Scale bar, 200 μm. Relative number and relative diameter of colonies are shown. Error bars, mean ± SD. Data were analyzed by two-tailed paired Student’s t-test. (C) Soft agar colony formation assay of sgCtrl- and sg*RELA*-transduced HepG2-TP53^R249S^ cell line. The images are representative of three independent experiments. Full-view images show the number of colonies, and the 4× magnification images indicated the size of the colonies. Scale bar, 200 μm. Relative number and relative diameter of colonies are shown. Error bars, mean ± SD. **P* ≤ 0.05; ***P* ≤ 0.01, data were analyzed by two-tailed paired Student’s t-test. (D) The tumor growth curves of HepG2-WT and HepG2-TP53^R249S^ transduced with sg*RELA* and sgCtrl (*n* = 5). Error bars, mean ± SD. ****P* ≤ 0.001, data were analyzed by two-way ANOVA with Tukey’s multiple comparison test. (E) The tumor weight statistics are shown. Error bars, mean ± SD. **P* ≤ 0.05; ****P* ≤ 0.001, data were analyzed by two-tailed unpaired Student’s t-test. (F) Representative proliferation curves for HepG2-WT or HepG2-MYC transduced with sg*RELA* and sgCtrl respectively. Error bars, mean ± SD. ****P* ≤ 0.001, data were analyzed by two-way ANOVA with Tukey’s multiple comparison test. (G) Soft agar colony formation assay of sgCtrl- and sg*RELA*-transduced HepG2 or HepG2-MYC cells. The images are representative of three independent experiments. Full-view images show the number of colonies, and the 4× magnification images indicate the size of the colonies. Scale bar, 200 μm. Relative diameter of colonies are shown. Error bars, mean ± SD. Data were analyzed by two-tailed paired Student’s t-test.


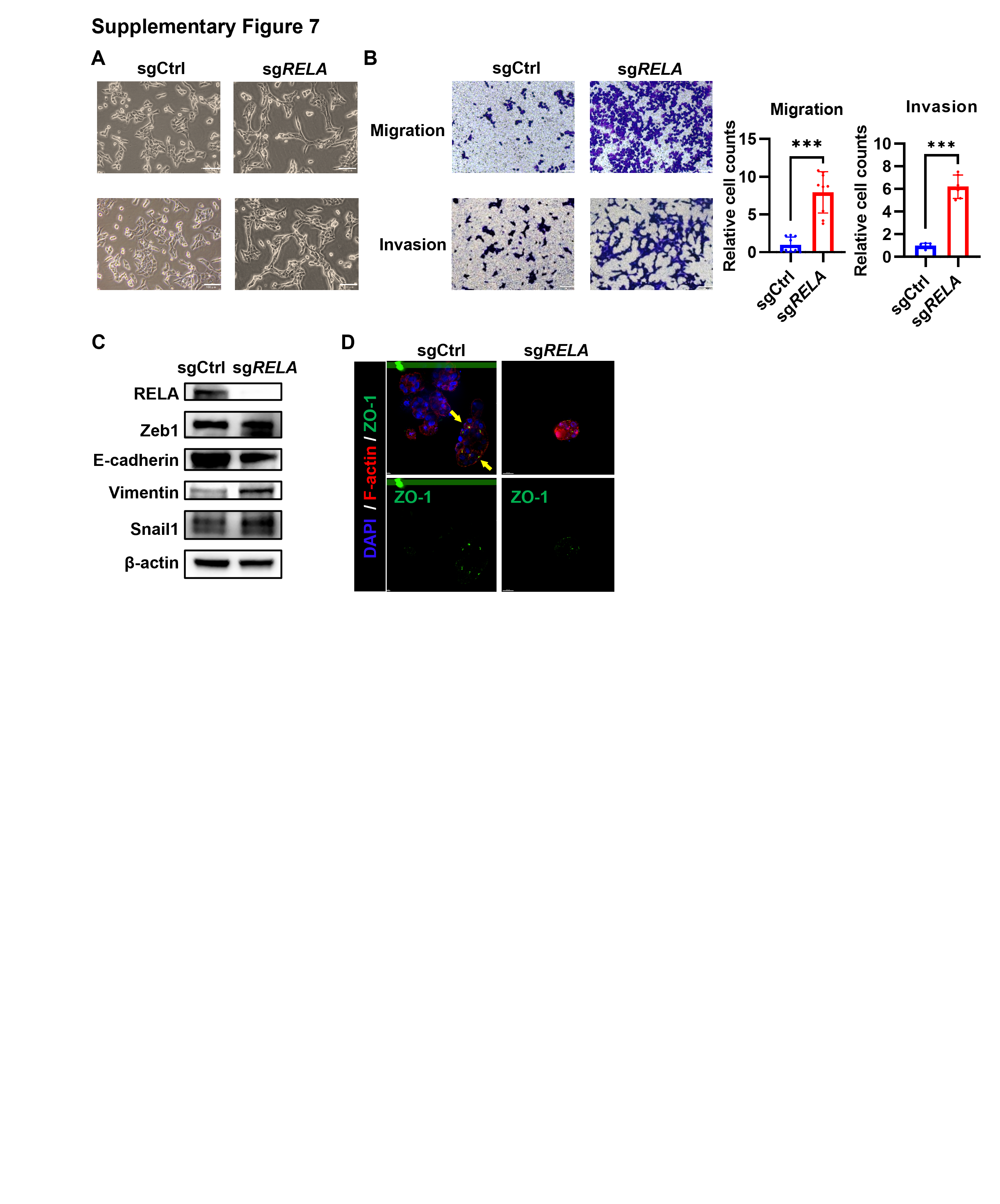


**Figure S7. RELA deficiency enhanced EMT program of MT-PHHs *in vitro*.**

(A) Representative images of MT-PHHs-sgCtrl and MT-PHHs-sg*RELA* cultured at a stable condition. Scale bars, 100 μm. (B) Cell migration (top) and cell invasion (bottom) were estimated using Transwell assay and quantified by relative counts of crystalline violet-stained cells. Error bars, mean ± SD. ****P* ≤ 0.001, data were analyzed by two-tailed paired Student’s t-test. Pictures of wells are representative of three independent experiments. (C) Western Blot analysis of protein levels of RELA, Zeb1, E-cadherin, vimentin, Snail1 in MT-PHHs-sgCtrl and MT-PHHs-sg*RELA*. (D) Representative images of immunofluorescence staining of MT-PHHs-sgCtrl and MT-PHHs-sg*RELA* formed organoids plated in 3D Matrigel-matrix and stained after 6 days for Zonula-occludens 1 (ZO-1, green), F-actin microfilaments using phalloidin (red) and nuclei using DAPI (blue). The yellow arrows represent the apico-basal polarity and formed hollow lumen. Scale bars, 100 μm.


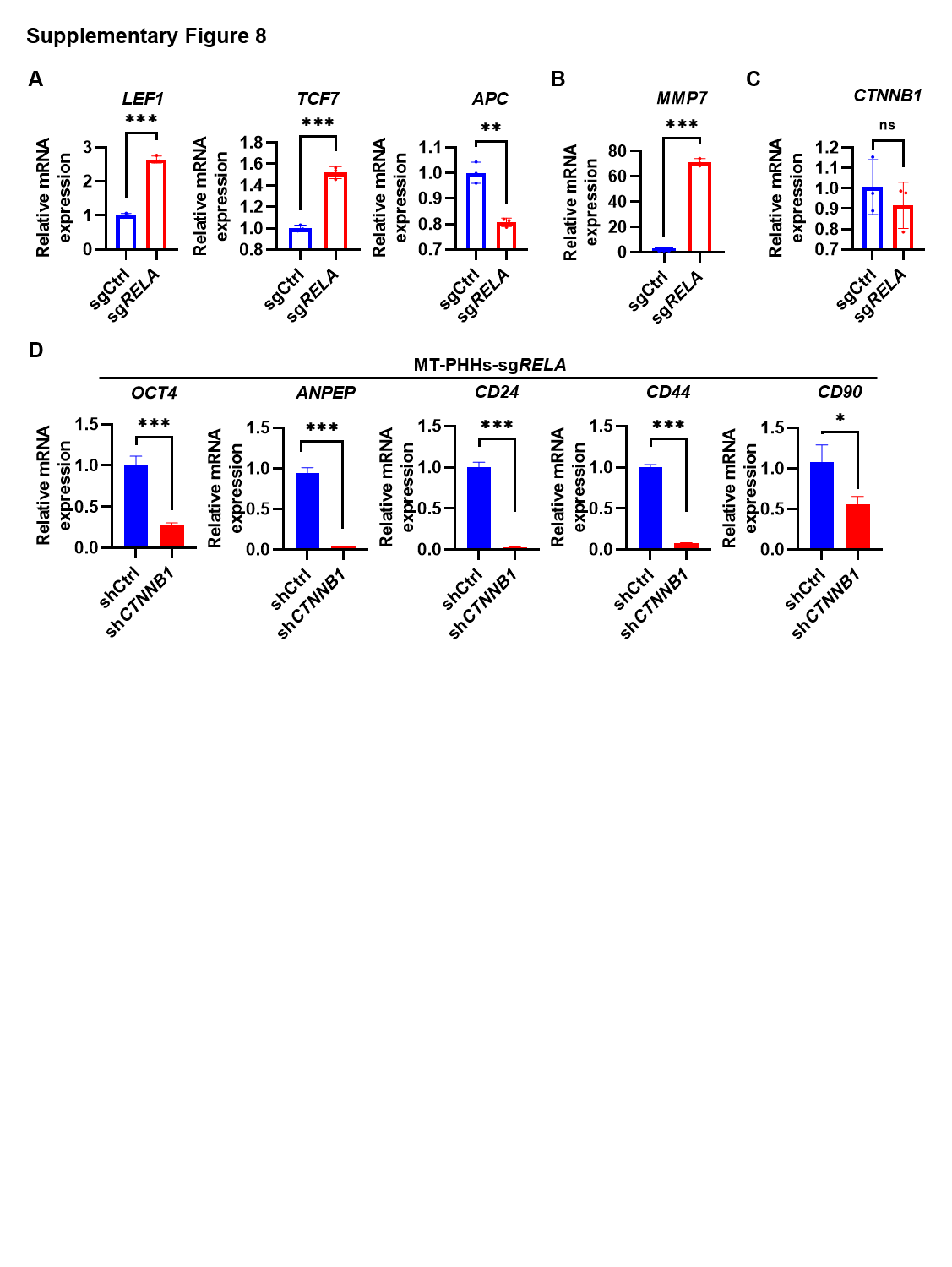


**Figure S8. The inhibition of β-catenin decreased cell stemness of MTR-PHHs.**

(A) Relative mRNA levels of *LEF1*, *TCF7* and *APC* in MT-PHHs-sgCtrl and MT-PHHs-sg*RELA*, based on quantitative RT-PCR. Error bars, mean ± SD. ***P* ≤ 0.01; ****P* ≤ 0.001, data were analyzed by two-tailed paired Student’s t-test. (B) Relative mRNA levels of *MMP7* in MT-PHHs-sgCtrl and MT-PHHs-sg*RELA*, based on quantitative RT-PCR. Error bars, mean ± SD. ****P* ≤ 0.001, data were analyzed by two-tailed paired Student’s t-test. (C) Relative mRNA levels of *CTNNB1* in MT-PHHs-sgCtrl and MT-PHHs-sg*RELA*, based on quantitative RT-PCR. Error bars, mean ± SD. ns > 0.05, data were analyzed by two-tailed paired Student’s t-test. (D) Relative mRNA levels of *OCT4*, *ANPEP*, *CD24*, *CD44* and *CD90* in shCtrl- and sh*CTNNB1*-transduced MT-PHHs-sg*RELA*, based on quantitative RT-PCR. Error bars, mean ± SD. **P* ≤ 0.05; ****P* ≤ 0.001, data were analyzed by two-tailed paired Student’s t-test.


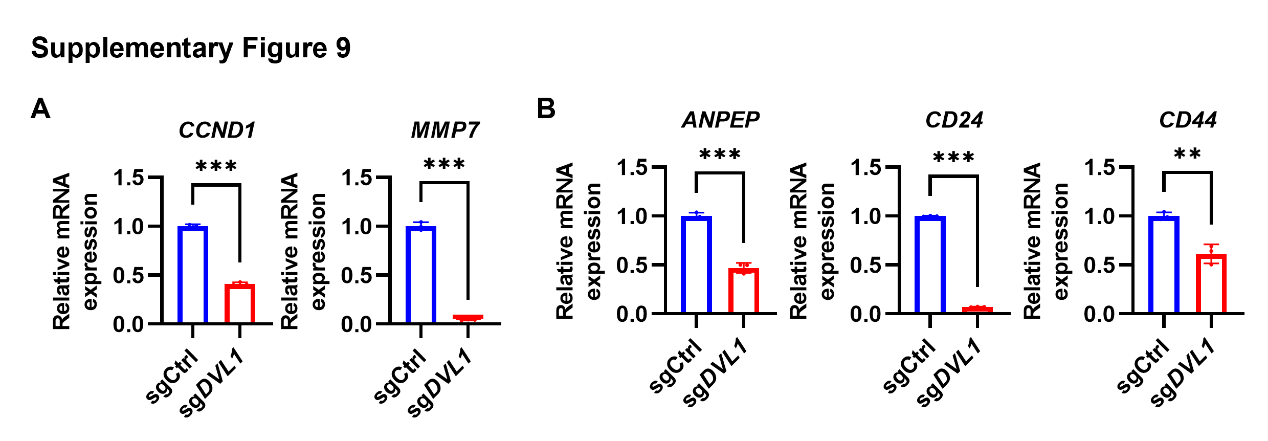


**Figure S9. The deletion of DVL1 decreased Wnt/β-catenin downstream target genes.**

(A) Relative mRNA levels of *CCND1* and *MMP7* in sgCtrl- or sg*DVL1*-transduced MT-PHHs-sg*RELA*, based on quantitative RT-PCR. Error bars, mean ± SD. ****P* ≤ 0.001, data were analyzed by two-tailed paired Student’s t-test. (B) Relative mRNA levels of *ANPEP*, *CD24* and *CD44* in sgCtrl- or sg*DVL1*-transduced MT-PHHs-sg*RELA*, based on quantitative RT-PCR. Error bars, mean ± SD. ***P* ≤ 0.01; ****P* ≤ 0.001, data were analyzed by two-tailed paired Student’s t-test.


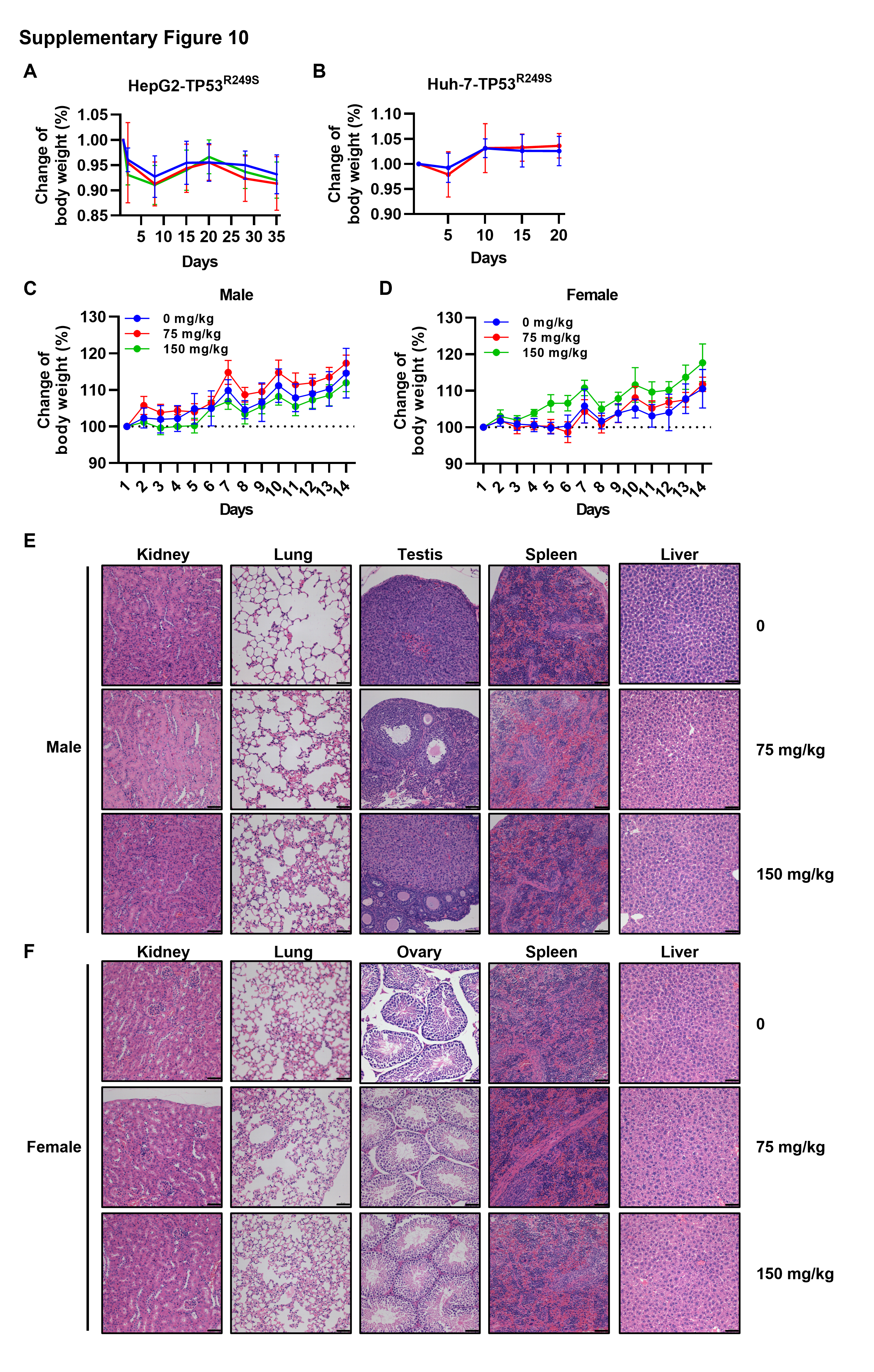


**Figure S10. BetA treatment showed no significant toxicity in mice.**

(A) Body weight changes (%) in HepG2-TP53^R249S^ xenograft mouse models following vehicle or BetA treatment (*n* = 6). (B) Body weight changes (%) in Huh-7-TP53^R249S^ xenograft mouse models following vehicle or BetA treatment (*n* = 6). (C) Body weight changes (%) in male mice following vehicle or BetA (75 mg/kg or 150 mg/kg) treatment (*n* = 5). (D) Body weight changes (%) in female mice following vehicle or BetA (75 mg/kg or 150 mg/kg) treatment (*n* = 5). (E) Representative images of H&E staining of kidney, lung, testis, spleen and liver tissues derived from male mice following vehicle or BetA (75 mg/kg or 150 mg/kg) treatment. Scale bar, 50 μm. (F) Representative images of H&E staining of kidney, lung, ovary, spleen and liver tissues derived from female mice following vehicle or BetA (75 mg/kg or 150 mg/kg) treatment. Scale bar, 50 μm.

**
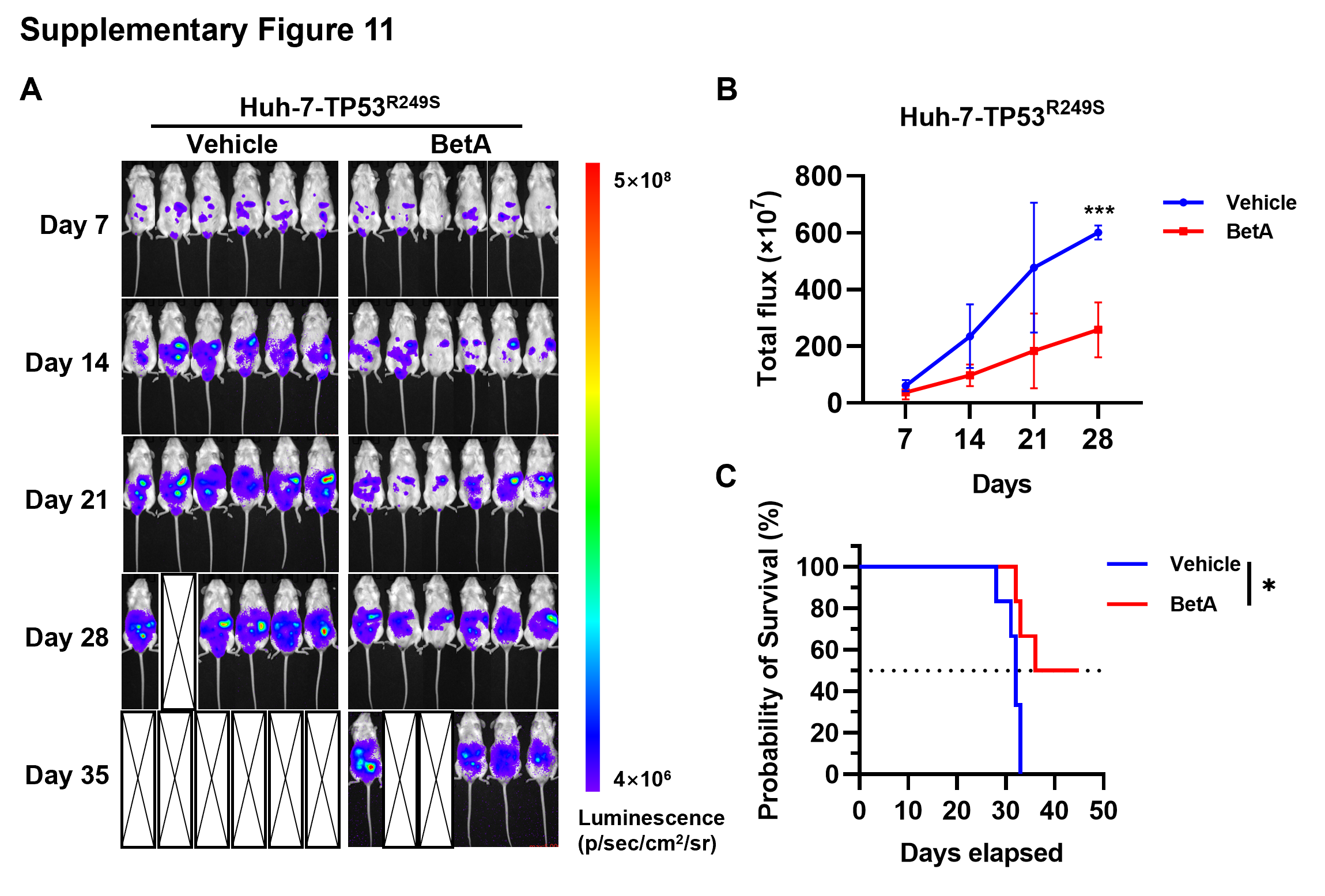
**

**Figure S11. BetA treatment exhibited anti-metastatic potential in TP53^R249S^-mutant liver cancer cells.**

(A) Bioluminescence signals of Huh-7-TP53^R249S^-indudced metastatic mouse model (*n* = 6) treated with vehicle or BetA (15 mg/kg) at indicated time points. The areas with cross represent mice mortality. (B) Total fluorescence intensity statistics of bioluminescence signals in (A) are shown. Error bars, mean ± SD. ****P* ≤ 0.001, data were analyzed by two-way ANOVA with Tukey's multiple comparison test. (C) Survival curves of mice in (A) treated with vehicle or BetA. **P* ≤ 0.05. The survival curves were analyzed using the Kaplan-Meier method, and statistical significance was determined by the log-rank test.

**Supplementary Tables**

**Table S1.**  **Clinical characteristics of 96 patients with HCC.**

| Variables |  |
| --- | --- |
| Age, years (median, range) | (60, 3-83) |
| Sex (male/ female) | (75/21) |
| Hepatitis B carrier(positive/negative) | (69/27) |
| Cirrhosis (yes/no) | (32/64) |
| AFP, ng/ml (<400/≥400) | (70/26) |
| Tumor size, cm (≤ 5/> 5) | (41/55) |
| UICC grade (Ⅰ-Ⅱ/Ⅲ-Ⅳ) | (34/62) |

**Table S2. Antibodies, sgRNAs and shRNAs used in this study.**

| Targets | Clone | Vendor | Catalog number | Application |
| --- | --- | --- | --- | --- |
| HLA | EMR8-5 | abcam | ab70328 | IHC |
| AFP | EPR9309 | Biolegend | ab169552 | IHC |
| Ki-67 | 8D5 | Cell Signaling Technology | 9449 | IHC |
| p21 | 12D1 | Cell Signaling Technology | 2947 | IHC |
| RELA | D14E12 | Cell Signaling Technology | 8242 | IHC &Western blot &IF |
| GFP | - | Proteintech | 50430-2-AP | IHC |
| F-actin | - | Yeasen | 40762ES75 | IF |
| E-cadherin | 24E10 | Cell Signaling Technology | 3195 | Western blot &IF |
| N-cadherin | D4R1H | Cell Signaling Technology | 13116 | Western blot &IF |
| ZO-1 | 1/ZO-1 (RUO) | B&D | 610967 | IF |
| Vimentin | D21H3 | Cell Signaling Technology | 5741 | Western blot |
| GAPDH | 1E6D9 | Proteintech | 60004-1 | Western blot |
| Zeb1 | E2G6Y | Cell Signaling Technology | 83243SF | Western blot |
| Snail1 | C15D3 | Cell Signaling Technology | 3879 | Western blot |
| β-catenin | D10A8 | Cell Signaling Technology | 8480S | Western blot |
| LEF1 | C12A5 | Cell Signaling Technology | 2230 | Western blot |
| Cyclin D1 | E3P5S | Cell Signaling Technology | 75463 | Western blot |
| OCT-4 | D7O5Z | Cell Signaling Technology | Ab188474 | Western blot |
| CD44 | 156-3C11 | Cell Signaling Technology | 3570T | Western blot |
| β-actin | AC004 | Abclonal | 3700 | Western blot |
| H3 | D1H2 | Cell Signaling Technology | 60932SF | Western blot |
| DVL1 | - | Proteintech | 27384-1-AP | Western blot |
| DVL3 | - | Proteintech | 13444-1-AP | Western blot |
| Phospho-RELA (Ser536) | 93H1 | Cell Signaling Technology | 3033 | Western blot |
| sg*RELA* | GGGAGATGCGCACTGTCCCT | | | Function |
| sg*RELA*-1 | CTGCTTGGGCTGCTCAATGA | | | Function |
| sg*RELA*-2 | CTAGAGGTGTATCCCCGGTC | | | Function |
| sg*NF2* | TGTCACTGTACGAGATGTTT | | | Function |
| sg*CSK* | CGATCACGCCCAGGAGCTGC | | | Function |
| sg*SGK3* | GAACTTTATAAACCTGTGGA | | | Function |
| sg*DPAGT1* | AGTATCGGGCGGAAGGGCTT | | | Function |
| sg*DCBLD2* | CGGAACAGCAATGACCTCAA | | | Function |
| sg*TOE1* | TGTAACGTTCCTCAATGCAC | | | Function |
| sg*LIMD1* | AGATTACCACAAGTAAGAAG | | | Function |
| sg*DVL1*-1 | GTACCTGGTCAAGCTGCCCG | | | Function |
| sg*DVL1*-2 | TCTTGGTCTCCGCCATGGCG | | | Function |
| sgCtrl-1 | TTGCGACGCTTAGCCTCCGT | | | Function |
| sgCtrl-2 | TTGAACGGGCCGCGGAAGCG | | | Function |
| sh*CTNNB1*-1-F | CGGGCATAACCTTTCCCATCATTTGGATCCAAATGATGGGAAAGGTTATGCTTTTTG | | | Function |
| sh*CTNNB1*-1-R | AATTCAAAAAGCATAACCTTTCCCATCATTTGGATCCAAATGATGGGAAAGGTTATGCCCGGTAC | | | Function |
| sh*CTNNB1*-2-F | CGGGCTGGTGGAATGCAAGCTTTTGGATCCAAAAGCTTGCATTCCACCAGCTTTTTG | | | Function |
| sh*CTNNB1*-2-R | AATTCAAAAAGCTGGTGGAATGCAAGCTTTTGGATCCAAAAGCTTGCATTCCACCAGCCCGGTAC | | | Function |

**Table S3. Primers for qRT-PCR or CUT&Tag-qPCR.**

| Genes | Forward primer 5’->3’ | Reverse primer 5’->3’ |
| --- | --- | --- |
| *CCNB1* | TTGGGGACATTGGTAACAAAGTC | ATAGGCTCAGGCGAAAGTTTTT |
| *CCND1* | GCTGCGAAGTGGAAACCATC | CCTCCTTCTGCACACATTTGAA |
| *CCNE1* | GCCAGCCTTGGGACAATAATG | CTTGCACGTTGAGTTTGGGT |
| *OCT4* | TCCACTTTGTATAGCCGCTGG | TGCATACACACAAACACAGCAA |
| *CD24* | CTCCTACCCACGCAGATTTATTC | AGAGTGAGACCACGAAGAGAC |
| *ANPEP* | TTCAACATCACGCTTATCCACC | AGTCGAACTCACTGACAATGAAG |
| *CD44* | CTGCCGCTTTGCAGGTGTA | CATTGTGGGCAAGGTGCTATT |
| *LEF1* | TGCCAAATATGAATAACGACCCA | GAGAAAAGTGCTCGTCACTGT |
| *TCF7* | TTGATGCTAGGTTCTGGTGTACC | CCTTGGACTCTGCTTGTGTC |
| *APC* | AAGCATGAAACCGGCTCACAT | CATTCGTGTAGTTGAACCCTGA |
| *MMP7* | ATGTGGAGTGCCAGATGTTGC | AGCAGTTCCCCATACAACTTTC |
| *CTNNB1* | CATCTACACAGTTTGATGCTGCT | GCAGTTTTGTCAGTTCAGGGA |
| *CD90* | ATCGCTCTCCTGCTAACAGTC | CTCGTACTGGATGGGTGAACT |
| *DVL1* | GAGGGTGCTCACTCGGATG | GTGCCTGTCTCGTTGTCCA |
| *DVL3* | TTCTTCAAGTCTATGGACGACGA | GAAGCATGGTAGCTTGGCATT |
| *DVL1* promoter1 (P1) | TCTGAAAGTACGTGGAGGACGGGAC | CCCATCTCCCCAAGACCTCCCTCCC |
| *DVL1* P2 | GGCTCCCCCCGCCCCACCCCACGAC | CTGGAAGGACTGGCGGCTGCCTGTC |
| *DVL1* P3 | GACCTTGGGCCGGTAAGCCAGGGTC | CAG GACCCTGGCCGACGGATGACTC |
| *ACTB* | GTCATTCCAAATATGAGATGCGTTG | TGCTATCACCTCCCCTGTGT |
| *GAPDH* | GGAGCGAGATCCCTCCAAAAT | GGCTGTTGTCATACTTCTCATGG |

The sequences of primers used in this study.
